# Multiplex Identification of Antigen-Specific T Cell Receptors Using a Combination of Immune Assays and Immune Receptor Sequencing

**DOI:** 10.1371/journal.pone.0141561

**Published:** 2015-10-28

**Authors:** Mark Klinger, Francois Pepin, Jen Wilkins, Thomas Asbury, Tobias Wittkop, Jianbiao Zheng, Martin Moorhead, Malek Faham

**Affiliations:** Adaptive Biotechnologies, South San Francisco, CA 94080, United States of America; University Paris Sud, FRANCE

## Abstract

Monitoring antigen-specific T cells is critical for the study of immune responses and development of biomarkers and immunotherapeutics. We developed a novel multiplex assay that combines conventional immune monitoring techniques and immune receptor repertoire sequencing to enable identification of T cells specific to large numbers of antigens simultaneously. We multiplexed 30 different antigens and identified 427 antigen-specific clonotypes from 5 individuals with frequencies as low as 1 per million T cells. The clonotypes identified were validated several ways including repeatability, concordance with published clonotypes, and high correlation with ELISPOT. Applying this technology we have shown that the vast majority of shared antigen-specific clonotypes identified in different individuals display the same specificity. We also showed that shared antigen-specific clonotypes are simpler sequences and are present at higher frequencies compared to non-shared clonotypes specific to the same antigen. In conclusion this technology enables sensitive and quantitative monitoring of T cells specific for hundreds or thousands of antigens simultaneously allowing the study of T cell responses with an unprecedented resolution and scale.

## Introduction

Assays enabling the identification and enumeration of antigen-specific T cells are critical tools in characterizing immune responses and harnessing T cell function for treatment of myriad diseases including cancer [1–5]. A variety of immune assays are currently used to quantitate antigen-specific T cells [6–11]. Multimer reagents including tetramers permit identification of antigen-specific T cells by direct binding of T cells to reagent. Other assays including ELISPOT, intracellular cytokine staining and proliferation assays, enumerate antigen-specific T cells based on detection of activation following stimulation of T cells with antigen.

Next generation sequencing of T cell receptor (TCR) repertoires has recently emerged as a new method to study immune responses [12–16]. This new tool enables identification and quantitation of all rearranged T cell antigen receptors, or clonotypes, contained within a sample. This allows the characterization of immune responses in a manner not possible previously and provides the sequences of the TCR as well as the dynamics and diversity of the repertoire. However, TCR sequence alone does not permit identification of the antigen a T cell recognizes. We previously demonstrated combining any of three immune assays (multimer binding, CD137 activation after stimulation, proliferation after stimulation) with TCR repertoire sequencing to identify TCRs specific for a particular index, or query antigen [17]. This technique has a number of advantages compared with traditional measures of antigen-specific T cells. The use of sequencing provides for high sensitivity and specificity amounting to single cell detection as well as quantitation accuracy in measuring the index antigen-specific T cell. In addition the diversity of TCR sequences specific to the index antigen can be elucidated. Finally, after identifying the index antigen-specific TCRs they can be tracked using DNA sequencing alone, therefore eliminating the need for live cells in subsequent samples.

A major limitation of this technique is that only one antigen can be assessed at a time. To assess several antigens the sample has to be split into different aliquots. Since many antigen-specific responses involve relatively low frequency T cells and the amount of sample available is finite there are limits to the number of antigens that can be assessed. An approach enabling assessment of numerous antigens simultaneously would overcome these limitations in addition to reducing effort and cost. Here we demonstrate an approach to identify T cells specific for each of 30 concurrently tested antigens. In principle this can be scaled-up to assess T cells specific for hundreds or thousands of antigens simultaneously.

## Results

### Technology description

**Fig 1:**
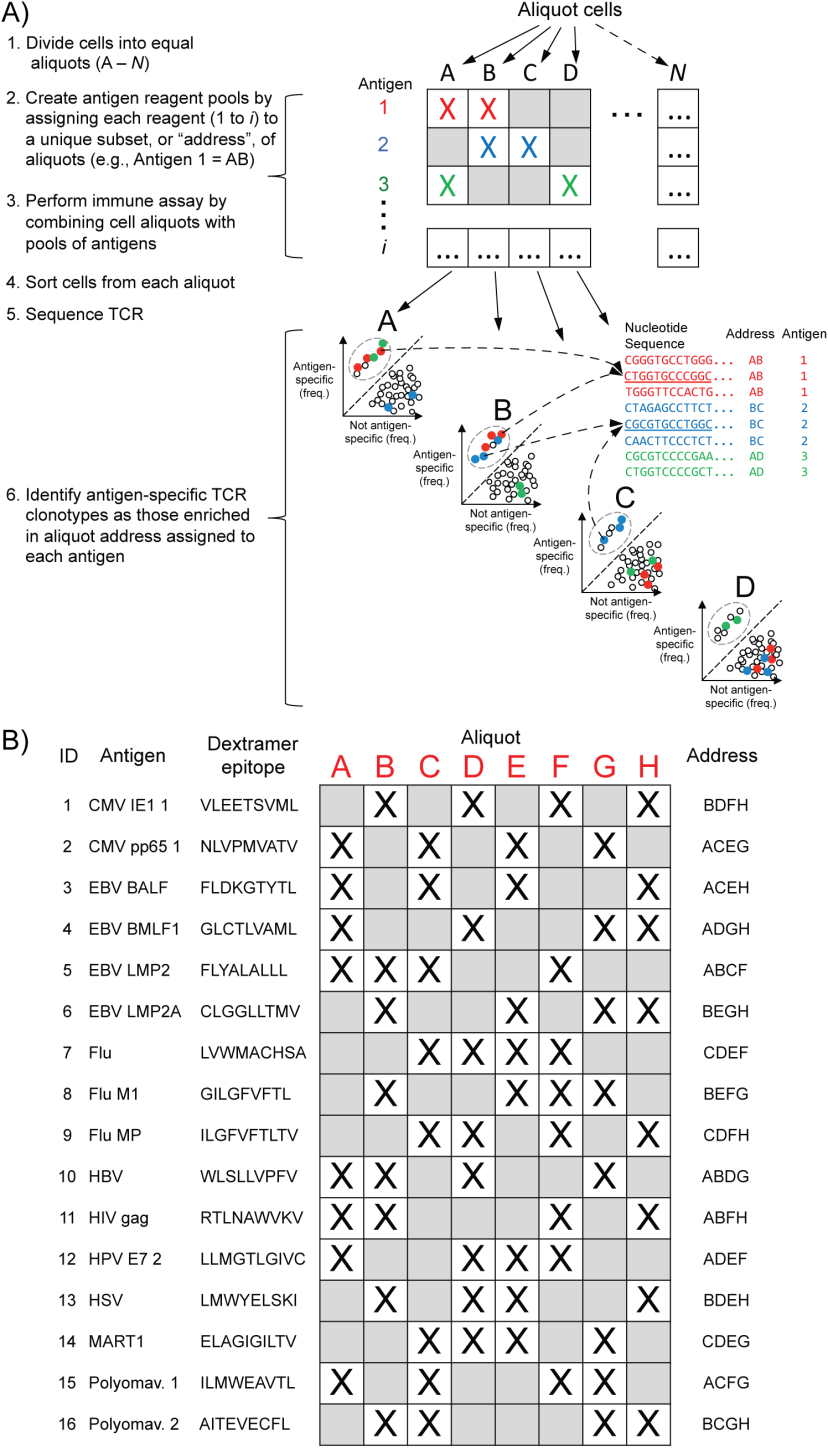
Overview of multiplex assay. **A)** MIRA assay outline: 1) Divide cells into equal aliquots (A to *N*), 2) Create immune assay antigen reagent pools by assigning each reagent (for example, dextramers or peptides; from a much larger set of antigens (1 to *i*) to a unique aliquot subset address, 3) Perform immune assay by combining each cell aliquot with the corresponding pool of antigens, 4) Sort T cells from each aliquot into two populations: antigen-specific and not antigen-specific, 5) Sequence TCR from all sorted populations, 6) Identify antigen-specific TCR clonotypes as those at higher frequency in the sorted antigen-specific population compared to the not antigen-specific population from the aliquot address assigned to each antigen reagent. **B)** MIRA set-up with dextramers. The PBMC sample is divided into an equal number of aliquots (A to H, indicated in red at top) matching the total number of dextramer, or antigen pools. Each dextramer is assigned to a unique subset, or “address”, of exactly four of eight pools as indicated in the right column. Individual dextramer assignments are indicated with an “X”. The CMV IE1 dextramer, for example, was assigned to pools B, D, F and H but not A, C, E or G.

The foundation of the approach to identify antigen-specific clonotypes is combining immune assays with sequencing of the TCRb repertoire. Cell sorting based on multimer binding, for example, enables separation of T cells into populations that are either antigen-specific or not (herein referred to as “positive” and “negative” populations, respectively). Both populations are sequenced and clonotypes specific to antigen are identified by virtue of enrichment in the positive population compared to the negative population (**S1 Fig**). To enable multiplexing different pools of antigens are combined together in such a way that each antigen is present in a unique subset of the pools (herein referred to as an “address”; **Fig 1A**). The PBMC sample is divided into a number of equal aliquots matching the total number of pools. Each cell aliquot is exposed to one of the antigen pools, positive and negative fractions are isolated, and sequencing is performed. Clonotypes specific to a test antigen would be enriched in pools containing the antigen but not in those that do not. The clonotype enrichment pattern reflects the antigen address and defines the antigen the clonotype is specific to.

The number of antigens that can be assessed at the same time is limited by the number of unique addresses that can be decoded. The greatest strength of this approach is that the number of addresses available increases exponentially with the number of pools created, thus enabling assessment of enormous numbers of antigens simultaneously. For example using 10 and 20 pools there are 1,024 and 1,048,576 possible addresses, respectively. In practice the number of pools would be smaller to ensure wider separation among addresses, but nevertheless the address space is massive with a manageable number of pools. We named the assay MIRA for ***M***ultiplexed ***I***dentification of T cell ***R***eceptor ***A***ntigen specificity.

### Multiplexing 16 dextramers to identify antigen-specific T cells

We first sought to identify clonotypes specific to 15 well-characterized viral antigens and 1 tumor-associated antigen from 4 healthy individuals known to carry an HLA-A*02 allele. We used HLA-A*02-restricted dextramers specific to these antigens. Eight pools of dextramers were constructed with each dextramer present in 4 of the 8 pools (see **Fig 1B** for dextramer assignments).

Each of the 8 pools of dextramers was then incubated with an aliquot of each donor PBMC sample. Dextramer positive (antigen-specific) and negative (not antigen-specific) populations were then isolated from each of the 8 aliquots by cell sorting. Nucleic acid was prepared from each of the 16 cell samples. TCRb was amplified and the repertoire sequenced using next generation sequencing. In addition we sequenced the TCRb repertoire from an unsorted PBMC sample from each donor.

From the sequencing data we then identified clonotypes whose frequencies were enriched in the sorted antigen-specific population of a particular antigen pool compared to the population from the same pool that is not antigen-specific. We then identified clonotypes that were enriched in 4 pools. Across all 4 donors 259 clonotypes were enriched in 4 out of 8 pools. We then identified the antigens these clonotypes were specific to by matching the pattern of the clonotype enrichment with the “address” of the 16 dextramers. The antigen relevant to each of the 259 identified clonotypes could be identified unambiguously.

**Fig 2:**
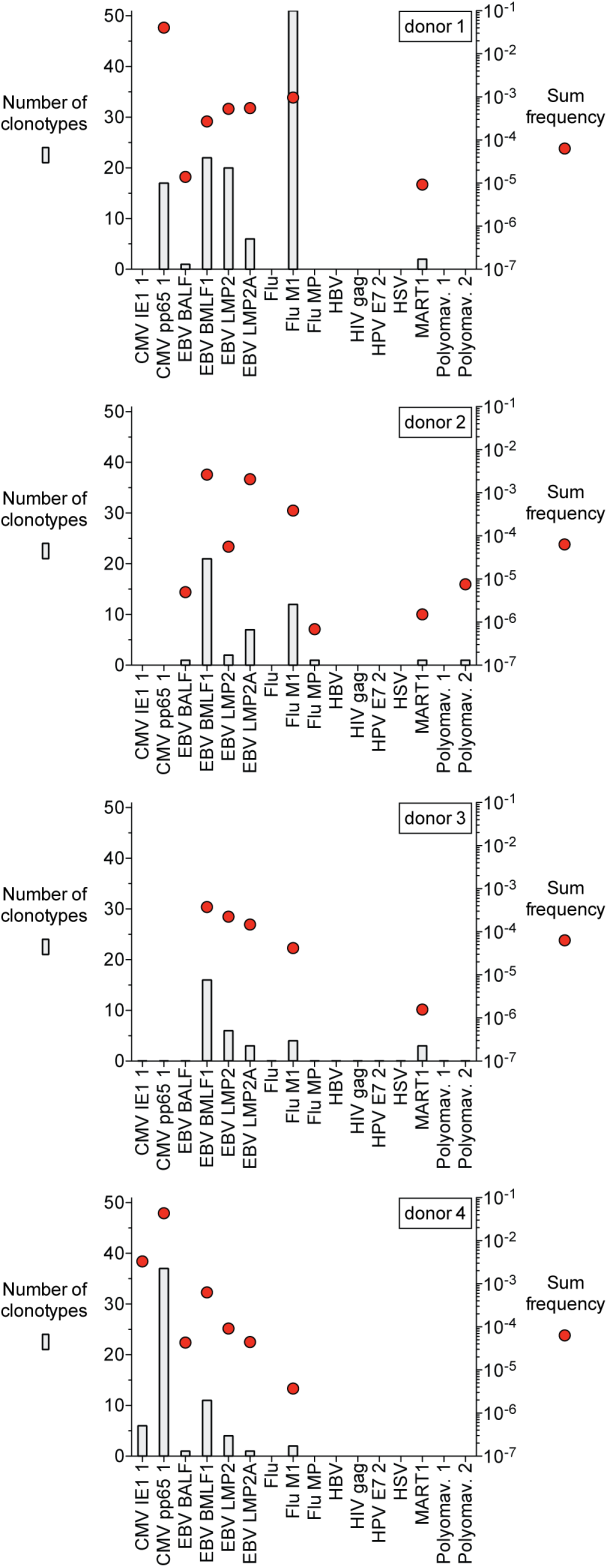
Number and frequency of antigen-specific clonotypes identified with dextramer-based MIRA. Plots show number (bars) and sum frequency (red circles) of antigen-specific clonotypes identified from each of four donors. Note donor 1 time point is the ‘month 2’ sample.

The breakdown of the 259 antigen-specific clonotypes from all 4 donors is shown in **Fig 2**and **S1 Table**. The number of clonotypes specific for a given dextramer varied widely both within and between individuals (range: 1 to 51). The sequence data from the unsorted sample was used to define the frequency of the identified antigen-specific clonotypes. The mean and median frequencies of antigen-specific T cell clonotypes across all donors was 1.9x10^-5^ and 1.6x10^-5^, respectively. The clonotype frequencies ranged from 6.9x10^-7^ to 2.7x10^-2^. The sum frequency of all clonotypes specific for a given dextramer within an individual also provided a quantitative measure of the response and varied depending on both the dextramer and individual (**Fig 2**; **S1 Table**).

### Validation of identified clonotypes by the type of matched addresses

There are 70 possible 4 of 8 address combinations. We only used 16 for the dextramers and therefore there were 54 “empty” addresses. If the pattern of enrichment was random we would expect the empty addresses to match the pattern of clonotype enrichment at the same rate as the 16 antigen addresses. In fact all 259 clonotypes identified in the donors matched 1 of the 16 dextramer addresses and none of the 54 unassigned addresses (Fisher's p-value 6x10^-48^).

### Repeatability of antigen-specific clonotype identification

The authenticity of the antigen-specific clonotypes identified were validated by repeating the assay with samples from the same individual (**S2 Fig**; **S2 Table**). 76 clonotypes were identified in both replicates and all were assigned the same antigen specificity. Clonotypes that were identified in one but not both replicates were low frequency and generally absent from one or more of the expected address subsets due to random fluctuation. Every discordant clonotype enriched in only 2 or 3 of 8 subsets was a partial match to the *bona fide* antigen address defined by the other replicate. We also identified antigen-specific clonotypes from PBMCs collected from blood drawn 2 months earlier from one of the donors (**S3 Fig**; **S3 Table**). 41 clonotypes were identified from both time points from this donor and all were assigned the same antigen specificity at both time points. As was observed with results from the replicate experiment, clonotypes enriched in one but not the other time point were low frequency and missing from one or more of the expected address subsets.

### Comparison with ELISPOT

**Fig 3:**
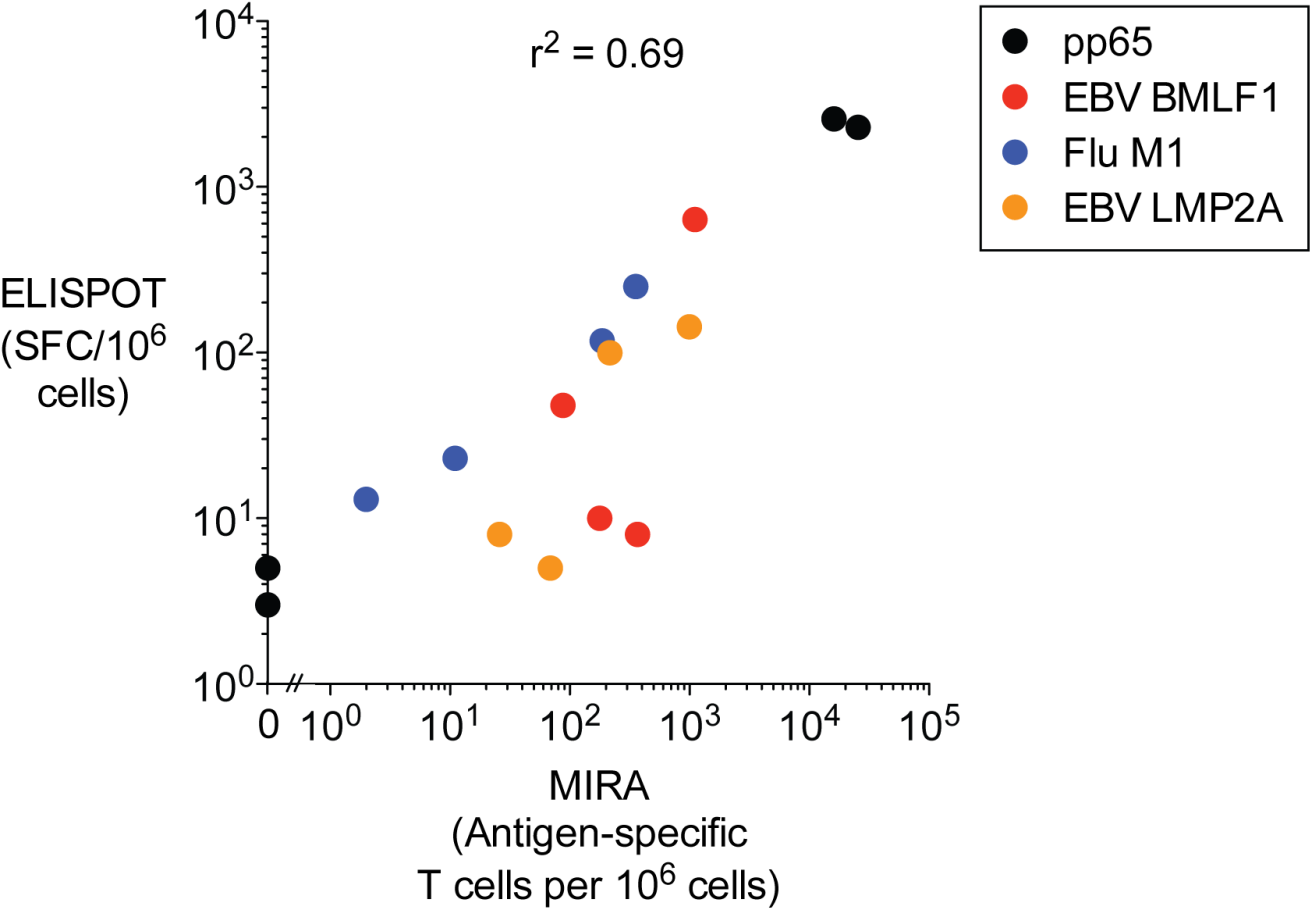
Comparison of MIRA and ELISPOT assay results. Plot of IFN-γ ELISPOT (y-axis; Spot Forming Cells per 10^6^ cells) and MIRA (x-axis; Antigen-specific T cells per 10^6^ cells) results for the set of four antigens assessed from each of four donors. The ‘antigen-specific T cells per 10^6^ cells’ values for each donor were calculated by summing frequencies of all clonotypes specific for a given antigen and dividing by the proportion of T cells in each donor PBMC sample based on sort gate frequencies.

ELISPOT results for 4 antigens were independently generated for each donor. ELISPOT measures the total number of antigen-specific T cells secreting a particular cytokine. If all antigen-specific T cells secrete the cytokine measured by ELISPOT then results would be analogous to the sum frequency of antigen-specific clonotypes identified by MIRA. We compared IFN-γ ELISPOT results with the sum frequency of antigen-specific clonotypes from each donor (**Fig 3**). There was a high correlation between results from both assays (r^2^ = 0.69) and MIRA readily detected antigen-specific clonotypes below 1 in 100,000 PBMCs, below estimates of the limit of detection for ELISPOT of ~4 spots per 100,000 PBMCs [18]. The few discordant measurements between MIRA and ELISPOT may be the result of a fraction of antigen-specific T cells not secreting IFN-γ.

### Assay variants

PBMCs from multiple donors can also be mixed and assessed simultaneously to identify antigen-specific clonotypes against a set of antigens with high accuracy. Antigen-specific clonotypes were identified in an experiment using a mixture of PBMCs from 3 donors (**S4 Table**). TCRb clonotype nucleotide sequences are sufficiently unique to enable assignment of a specific clonotype to a donor and each antigen-specific clonotype identified was derived from a single individual upon comparison to clonotypes obtained from the independently sequenced PBMC sample from each donor. Also, since we previously performed MIRA on each donor individually (**Fig 2**) we could compare antigen-specificity assignments in the mixture of donors to those derived from individual donors. 181 of the antigen-specific clonotypes identified from the mixture of PBMCs matched an antigen-specific clonotype from 1 of the 3 donors and in all cases the antigen specificity assignment was the same in both the individual and mixed PBMC experiments. We also repeated the assay with the same mixture of donors to compare antigen-specific clonotype detection between DNA and RNA isolated from the same cells. Multiple copies of RNA per cell is expected to lead to better sensitivity and as expected 50% more antigen-specific clonotypes were identified with RNA compared to DNA (**S5 Table**).

### Multiplexing 30 peptides for identification of antigen-specific clonotypes using peptide stimulation of PBMCs

**Fig 4:**
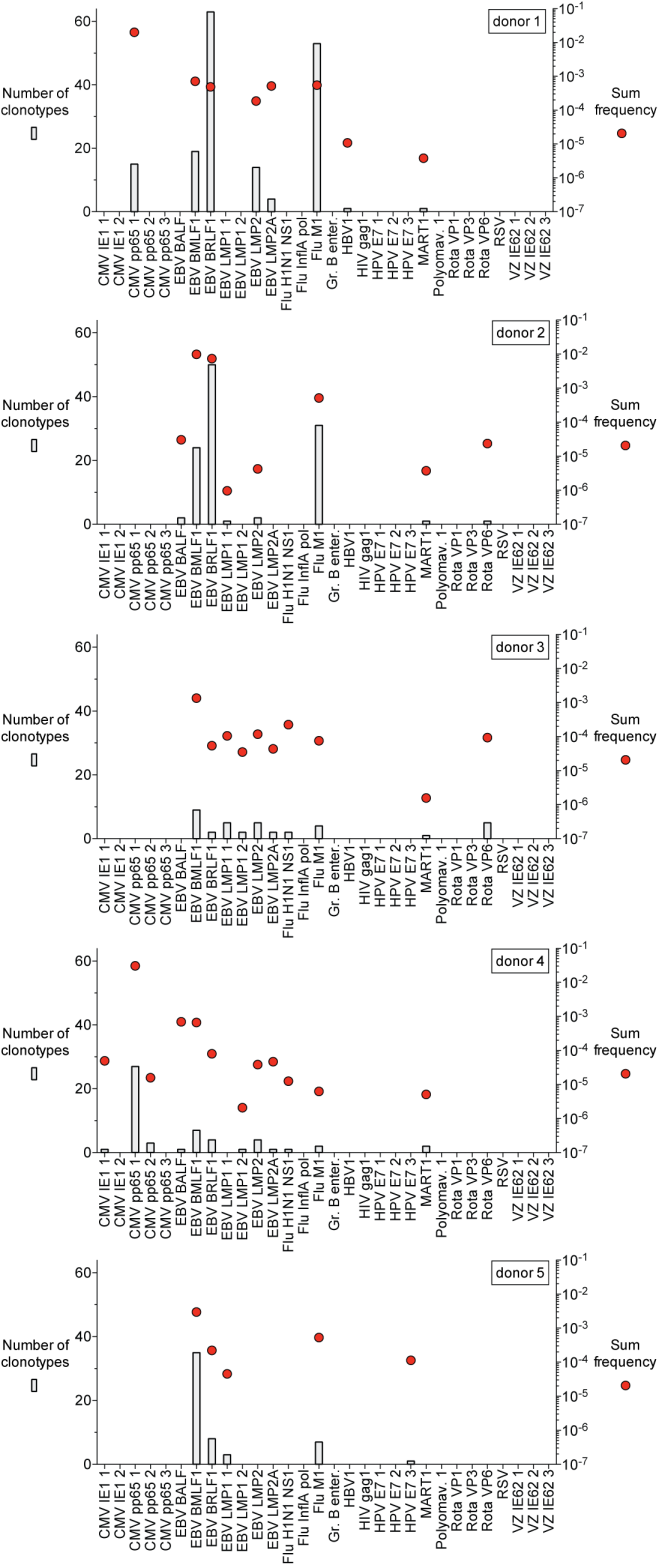
Number and frequency of antigen-specific clonotypes identified with peptide-based MIRA. Plots show number (bars) and sum frequency (red circles) of antigen-specific clonotypes identified with peptides from each of five donors. The donor 1 sample used in this experiment was from the earlier of the two time points used from this donor (‘month 0’).

Generating multimers for a large number of antigens can be cumbersome. We therefore modified the multiplex assay by incorporating PBMC stimulation with a peptide pool (instead of incubation with pools of dextramers) and an indirect, or functional T cell activation read-out. Specifically we sorted CD8 T cells that were antigen-specific and not antigen-specific based on CD137 expression following short-term peptide stimulation. In these experiments we sought to identify antigen-specific clonotypes against a set of 30 peptide antigens in different individuals. Ten different pools of peptide antigens were constructed in such a way that each peptide was present in a unique subset, or address, of 5 of the 10 pools (**S4 Fig**). We then stimulated PBMCs from each of 5 donors using these 10 pools of peptide antigens (Note that donors 1 to 4 are the same individuals as used in the dextramer-based MIRA results outlined previously). We identified a total of 427 antigen-specific clonotypes in the 5 donors as summarized in **Fig 4**and **S6 Table**. Since 9 of the 30 peptides used were the same as what was engineered in 9 of the dextramers used in the results outlined above we could compare results from both types of assays. 115 clonotypes were identified in both the dextramer- and peptide-based MIRA assays and in each case the antigen specificity assignment was identical.

### Identification of multiple highly similar antigen-specific clonotypes within and between individuals

We reasoned that another way to validate the authenticity of the antigen-specific clonotypes would be to identify identical or highly similar clonotype protein sequences within or between individuals. For the peptide-based MIRA assay we identified all the antigen-specific clonotype protein sequences that were encoded by multiple nucleotide sequences within and/or between individuals. Altogether 381 distinct clonotype protein sequences were encoded by the antigen-specific clonotype nucleotide sequences from all 5 donors. While most of these clonotype protein sequences were encoded by a single antigen-specific clonotype nucleotide sequence, 21 were encoded by multiple distinct nucleotide sequences within and/or between donors (**Table 1**). In 21 of 21 cases the antigen specificities of the multitude of clonotype nucleotide sequences corresponding to each of the clonotype protein sequences were identical. Within any donor up to 5 distinct nucleotide sequences encoded each of these 21 clonotype protein sequences. Interestingly 1 clonotype protein sequence (Flu M1-specific) was identified in 5 of 5 donors. Additionally 11 of these clonotype protein sequences encoded by multiple nucleotide sequences were identified independently with dextramers (Table 1 - third column) and in each case the determined antigen-specificity was the same.

**Table 1:** Clonotype protein sequences encoded by multiple nucleotide sequences from peptide-based MIRA.

Clonotype protein sequence*	Antigen	CDR3 also identified with dextramer-based MIRA**	Matches published CDR3/Antigen match	Donor	Clonotype nucleotide sequence
TAFYL**CASSGRSTDTQYF**GPGT	M1	Yes (M1)	Not found	1	CGGGTGCCTGGGCCAAAATACTGCGTATCTGTGCTCCGACCAGAGCTGGCACAGAGATAGAAAGCTGTCG
					CGGGTGCCTGGGCCAAAATACTGCGTATCTGTGCTCCTCCCGCTACTGGCACAGAGATAGAAAGCTGTCG
					CGGGTGCCTGGGCCAAAATACTGCGTATCTGTGGACCGCCCACTACTGGCACAGAGATAGAAAGCTGTCG
					CGGGTGCCTGGGCCAAAATACTGCGTATCTGTGCTCCTTCCGCTACTGGCACAGAGATAGAAAGCTGTCG
TAFYL**CASSIRSSYEQYF**GPGT	M1	Yes (M1)	Match/Yes	1	CTGGTGCCCGGCCCGAAGTACTGCTCGTAGGAGCTACGGATACTACTGGCACAGAGATAGAAAGCTGTCG
					CTGGTGCCCGGCCCGAAGTACTGCTCGTAGGAGCTCCTGATACTACTGGCACAGAGATAGAAAGCTGTCG
					CTGGTGCCCGGCCCGAAGTACTGCTCGTAGGAGCTGCGGATACTACTGGCACAGAGATAGAAAGCTGTCG
					CTGGTGCCCGGCCCGAAGTACTGCTCGTAGGAGCTTCGAATACTACTGGCACAGAGATAGAAAGCTGTCG
				2	CTGGTGCCCGGCCCGAAGTACTGCTCGTAGGAGCTCCGAATACTACTGGCACAGAGATAGAAAGCTGTCG
					CTGGTGCCCGGCCCGAAGTACTGCTCGTAGGAGCTCCGGATACTACTGGCACAGAGATAGAAAGCTGTCG
					CTGGTGCCCGGCCCGAAGTACTGCTCGTAGGAGCTCCTGATACTACTGGCACAGAGATAGAAAGCTGTCG
					CTGGTGCCCGGCCCGAAGTACTGCTCGTAGGAGCTTCGGATACTACTGGCACAGAGATAGAAAGCTGTCG
					CTGGTGCCCGGCCCGAAGTACTGCTCGTAGGAGGACCTAATACTACTGGCACAGAGATAGAAAGCTGTCG
				3	CTGGTGCCCGGCCCGAAGTACTGCTCGTAGGAGCTTCTTATACTACTGGCACAGAGATAGAAAGCTGTCG
					CTGGTGCCCGGCCCGAAGTACTGCTCGTAGGAACTCCGTATACTACTGGCACAGAGATAGAAAGCTGTCG
				4	CTGGTGCCCGGCCCGAAGTACTGCTCGTAGGAGCTCCGGATACTACTGGCACAGAGATAGAAAGCTGTCG
				5	CTGGTGCCCGGCCCGAAGTACTGCTCGTAGGAGCTCCTTATACTACTGGCACAGAGATAGAAAGCTGTCG
TAFYL**CASSIRSAYEQYF**GPGT	M1	Yes (M1)	Not found	1	CTGGTGCCCGGCCCGAAGTACTGCTCGTAGGCAGAGCGGATACTACTGGCACAGAGATAGAAAGCTGTCG
				5	CTGGTGCCCGGCCCGAAGTACTGCTCGTAGGCCGATCTAATACTACTGGCACAGAGATAGAAAGCTGTCG
				2	CTGGTGCCCGGCCCGAAGTACTGCTCGTAGGCGCTCCGGATACTACTGGCACAGAGATAGAAAGCTGTCG
TAFYL**CASSMRSAYEQYF**GPGT	M1	No	Not found	1	CTGGTGCCCGGCCCGAAGTACTGCTCGTAGGCACTCCGCATACTACTGGCACAGAGATAGAAAGCTGTCG
				3	CTGGTGCCCGGCCCGAAGTACTGCTCGTAGGCCGATCGCATACTACTGGCACAGAGATAGAAAGCTGTCG
TAFYLCASSPRSTDTQYFGPGT	M1	No	Not found	1	CGGGTGCCTGGGCCAAAATACTGCGTATCTGTGCTACGTGGGCTACTGGCACAGAGATAGAAAGCTGTCG
					CGGGTGCCTGGGCCAAAATACTGCGTATCTGTGCTGCGGGGACTACTGGCACAGAGATAGAAAGCTGTCG
				2	CGGGTGCCTGGGCCAAAATACTGCGTATCTGTGCTCCGAGGACTACTGGCACAGAGATAGAAAGCTGTCG
TAFYL**CASSIRSTDTQYF**GPGT	M1	Yes (M1)	Not found	1	CGGGTGCCTGGGCCAAAATACTGCGTATCTGTGCTCCGGATACTACTGGCACAGAGATAGAAAGCTGTCG
				2	CGGGTGCCTGGGCCAAAATACTGCGTATCTGTGCTACGAATACTACTGGCACAGAGATAGAAAGCTGTCG
					CGGGTGCCTGGGCCAAAATACTGCGTATCTGTGCTCCGTATACTACTGGCACAGAGATAGAAAGCTGTCG
					CGGGTGCCTGGGCCAAAATACTGCGTATCTGTGCTTCGGATACTACTGGCACAGAGATAGAAAGCTGTCG
TAFYL**CASSIRSTGELF**FGEGS	M1	No	Not found	2	CTAGAGCCTTCTCCAAAAAACAGCTCCCCGGTACTCCGAATACTACTGGCACAGAGATAGAAAGCTGTCG
					CTAGAGCCTTCTCCAAAAAACAGCTCCCCGGTCGATCTGATACTACTGGCACAGAGATAGAAAGCTGTCG
TAFYL**CASSVRSSYEQYF**GPGT	M1	No	Not found	1	CTGGTGCCCGGCCCGAAGTACTGCTCGTAGGAGCTCCTGACCGAACTGGCACAGAGATAGAAAGCTGTCG
					CTGGTGCCCGGCCCGAAGTACTGCTCGTAGGAGGATCGTACACTACTGGCACAGAGATAGAAAGCTGTCG
				2	CTGGTGCCCGGCCCGAAGTACTGCTCGTAGGAGGATCTGACGCTACTGGCACAGAGATAGAAAGCTGTCG
YRCASSLAPGATNEKLFFGSGT	pp65	Yes (pp65)	Not found	4	TGGGTTCCACTGCCAAAAAACAGTTTTTCATTAGTTGCACCTGGCGCTAAGCTGCTGGCACAGCGATACA
					TGGGTTCCACTGCCAAAAAACAGTTTTTCATTAGTTGCCCCAGGGGCTAAGCTGCTGGCACAGCGATACA
YR**CASSLAPGTTNEKLF**FGSGT	pp65	Yes (pp65)	Match/Yes	4	TGGGTTCCACTGCCAAAAAACAGTTTTTCATTAGTTGTCCCAGGCGCTAAGCTGCTGGCACAGCGATACA
				1	TGGGTTCCACTGCCAAAAAACAGTTTTTCATTAGTTGTGCCGGGTGCTAAGCTGCTGGCACAGCGATACA
VYF**CASSYQTGAAYGYTF**GSGT	pp65	Yes (pp65)	Match/Yes	1	CTGGTCCCCGAACCGAAGGTGTAGCCATAGGCAGCCCCTGTCTGGTAACTGCTGGCACAGAAGTACACAG
				4	CTGGTCCCCGAACCGAAGGTGTAGCCATAGGCTGCCCCAGTCTGGTAACTGCTGGCACAGAAGTACACAG
SGVYFCASSQSPGGTQYFGPGT	BMLF	No	Match/Yes	5	CGCGTGCCTGGCCCGAAGTACTGGGTCCCCCCTGGGGATTGGCTGCTGGCACAGAAATAAACTCCAGAAT
				4	CGCGTGCCTGGCCCGAAGTACTGGGTCCCTCCTGGGGATTGGCTGCTGGCACAGAAATAAACTCCAGAAT
SSFYICSARDQTGNGYTFGSGT	BMLF	Yes (BMLF)	Match/Yes	2	CTGGTCCCCGAACCGAAGGTGTAGCCATTACCAGTCTGGTCTCTAGCACTGCAGATGTAGAAGCTGCTGT
				3	CTGGTCCCCGAACCGAAGGTGTAGCCATTCCCTGTCTGATCTCTAGCACTGCAGATGTAGAAGCTGCTGT
SFYI**CSARDRGIGNTIYF**GEGS	BMLF	Yes (BMLF)	Not found	3	CAACTTCCCTCTCCAAAATATATGGTGTTTCCAATCCCCCTGTCCCTAGCACTGCAGATGTAGAAGCTGC
				4	CAACTTCCCTCTCCAAAATATATGGTGTTTCCGATCCCCCTGTCCCTGGCACTGCAGATGTAGAAGCTGC
SSFYI**CSARDRTGNGYTF**GSGT	BMLF	No	Match/Yes	2	CTGGTCCCCGAACCGAAGGTGTAGCCATTCCCTGTCCGATCTCTAGCACTGCAGATGTAGAAGCTGCTGT
				5	CTGGTCCCCGAACCGAAGGTGTAGCCATTCCCTGTCCTATCCCTAGCACTGCAGATGTAGAAGCTGCTGT
SSFYI**CSARDRVGNTIYF**GEGS	BMLF	No	Match/Yes	5	CAACTTCCCTCTCCAAAATATATGGTGTTTCCAACGCGATCTCTAGCACTGCAGATGTAGAAGCTGCTGT
					CAACTTCCCTCTCCAAAATATATGGTGTTTCCCACCCTGTCCCTAGCACTGCAGATGTAGAAGCTGCTGT
				2	CAACTTCCCTCTCCAAAATATATGGTGTTTCCGACCCTGTCCCTAGCACTGCAGATGTAGAAGCTGCTGT
SSFYI**CSARVGVGNTIYF**GEGS	BMLF	No	Match/Yes	5	CAACTTCCCTCTCCAAAATATATGGTGTTTCCAACCCCCACCCGGGCACTGCAGATGTAGAAGCTGCTGT
					CAACTTCCCTCTCCAAAATATATGGTGTTTCCGACCCCTACTCTAGCACTGCAGATGTAGAAGCTGCTGT
SIYL**CSVGTGGTNEKLF**FGSGT	BMLF	Yes (BMLF)	Match/Yes	1	TGGGTTCCACTGCCAAAAAACAGTTTTTCATTAGTTCCCCCCGTCCCTACGCTGCAGAGATATATGCTGC
					TGGGTTCCACTGCCAAAAAACAGTTTTTCATTAGTTCCCCCTGTGCCCACGCTGCAGAGATATATGCTGC
				4	TGGGTTCCACTGCCAAAAAACAGTTTTTCATTAGTGCCCCCTGTCCCGACGCTGCAGAGATATATGCTGC
				5	TGGGTTCCACTGCCAAAAAACAGTTTTTCATTAGTTCCCCCTGTCCCAACGCTGCAGAGATATATGCTGC
				3	TGGGTTCCACTGCCAAAAAACAGTTTTTCATTAGTTCCCCCTGTCCCAACGCTGCAGAGATATATGCTGC
MYL**CASSSLRGSNQPQHF**GDGT	BRLF	not tested	Not found	1	CGAGTCCCATCACCAAAATGCTGGGGCTGATTGCTCCCCCGCAGGGAACTGCTGGCACAGAGGTACATAG
					CGAGTCCCATCACCAAAATGCTGGGGCTGATTGCTTCCCCTGAGGCTACTGCTGGCACAGAGGTACATAG
LYL**CASSQEGGGGYEQYF**GPGT	LMP1	not tested	Not found	3	CTGGTGCCCGGCCCGAAGTACTGCTCGTACCCGCCTCCCCCTTCTTGGCTGCTGGCACAGAGATACAGGG
					CTGGTGCCCGGCCCGAAGTACTGCTCGTAGCCCCCTCCCCCCTCTTGGCTGCTGGCACAGAGATACAGGG
				4	CTGGTGCCCGGCCCGAAGTACTGCTCGTATCCCCCCCCGCCTTCTTGGCTGCTGGCACAGAGATACAGGG
DSAVYL**CASSLGGYEQYF**GPGT	LMP2	Yes (LMP2)	Not found	1	CTGGTGCCCGGCCCGAAGTACTGCTCGTAGCCGCCGAGGCTGCTGGCACAGAGATACACGGCCGAGTCCT
					CTGGTGCCCGGCCCGAAGTACTGCTCGTATCCTCCTAAGCTGCTGGCACAGAGATACACGGCCGAGTCCT

M1, Flu M1; pp65, CMV pp65; BMLF, EBV BMLF1; BRLF, EBV BRLF1; LMP1, EBV LMP1 2; LMP2, EBV LMP2A

* CDR3 sequence in bold

** When also identified with dextramer-based MIRA, antigen-specificity is indicated in parentheses

We then identified all the antigen-specific clonotypes that were very similar to each other and differed by a single amino acid. Fifteen clonotype protein sequence clusters, in which the constituents of each cluster differed by a single amino acid, were identified within and between donors using the peptide-based MIRA assay. In every case the antigen specificities for the constituents within a cluster were identical (**S7 Table**). The assignment of identical antigen specificities to identical or highly similar clonotype protein sequences within and across donors further validates the authenticity of antigen-specific clonotype discovery with MIRA.

### Comparison with antigen-specific clonotypes identified in the literature

We also queried the CDR3 protein sequences from the antigen-specific clonotypes identified with peptide-based MIRA against a set of published CDR3 amino acid sequences. 15 of the antigen-specific clonotypes identified matched a query sequence from a set of 334 published HLA-A*02-restricted CMV, EBV and Influenza TCRb CDR3 amino acid sequences [19–32]. Nine of these were found within the set of clonotype protein sequences in **Table 1**(fourth column). The antigen specificity determined by MIRA for all 15 CDR3 amino acid sequences matched the previously published specificity.

### Prediction of clonotype antigen specificity in individuals based on MIRA antigen assignment in index individuals

Since different TCRa sequences can affect antigen specificity of TCRs with the same TCRb we wanted to explore whether the antigen specificity of a TCRb clonotype protein sequence in one individual is predictive of antigen specificity when that sequence is found in another individual. We started with all the clonotypes deemed specific to a tested antigen in at least one “index” of the 5 donors tested by peptide-based MIRA. We then asked if any of these clonotype protein sequences were present in PBMCs from any other donors. Altogether we found 122 sequences in these individuals matching an antigen-specific query sequence from an index donor. We then assessed the enrichment “behavior” of these clonotypes in the sorted positive (*i*.*e*., antigen-specific) fraction of each of the 10 multiplex aliquots in the queried individual. We reasoned that if the clonotype protein sequence from the queried individual exhibited the same antigen specificity as in the index donor it should be preferentially enriched in pools of the respective antigen address. Enrichment of these clonotypes in pools of the respective index antigen address compared to unassigned pools was much greater than expected by chance (p-value range: 0.007 to <10^−15^, depending on the comparison; **S8 Table**).

We then estimated the fraction of clonotypes whose antigen specificity could be predicted based on MIRA antigen assignment in another individual. We used a conservative assumption that the only reason a clonotype was not enriched in a pool of the respective expected address was that it was absent from the pool (*i*.*e*., missing from the positive and negative fractions from that pool). In 112/122 (92%) cases the clonotype was enriched in every pool of the expected address when a cell was present in the pool. We therefore concluded that for the tested antigens in HLA-A*02 positive individuals, identification of an antigen-specific clonotype in an index individual allowed prediction of antigen specificity of the same clonotype sequence when identified in another individual.

We also reasoned that if the sequences are specific to the tested antigens in the context of HLA-A*02 then these antigen-specific clonotypes were more likely to be present in HLA-A*02-positive compared to HLA-A*02-negative individuals. Therefore we sequenced the TCRb repertoire from 8 other HLA-A*02-positive and 6 HLA-A*02-negative individuals, none of which had been tested with MIRA. We then asked how many of the antigen-specific clonotypes from the dextramer- or peptide-based MIRA assays from the 5 index individuals were present in each of the new untested individuals. We found an average of 14 and 2 clonotypes in the HLA-A*02 positive and negative individuals, respectively (p-value 0.0094) (**S5 Fig**). This is consistent with the notion that in the majority of cases when clonotypes are identified as antigen-specific in an index individual with MIRA and that sequence is then observed in another individual with the same HLA, that clonotype is specific to the same antigen.

### Complexity and frequency of shared clonotypes

**Fig 5:**
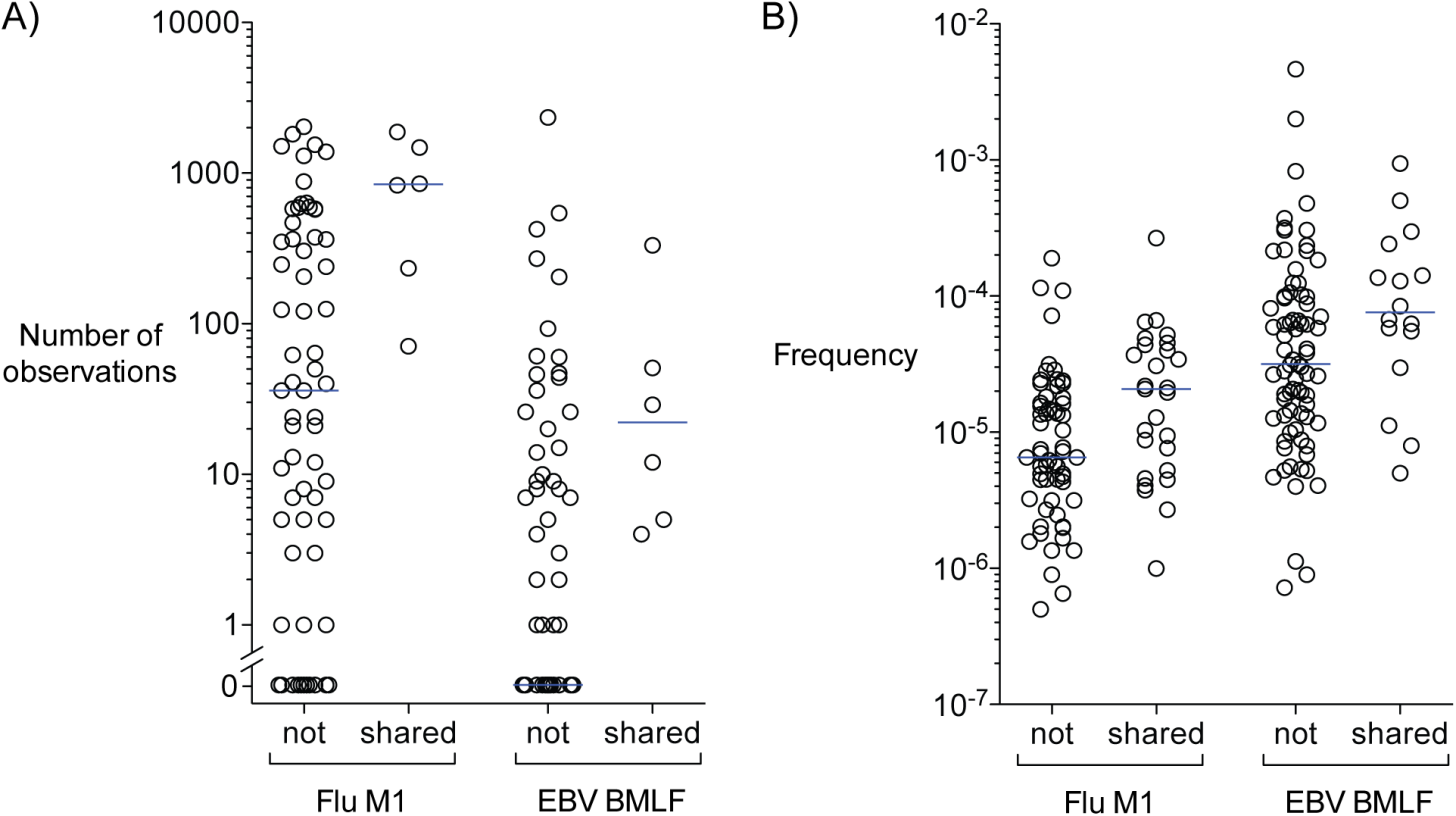
Shared antigen-specific clonotype protein sequences are simpler to generate and more abundant than those that are not shared between individuals. **A)** Plot of the relative abundance of each shared (identified in more than one individual) and not shared Flu M1- and EBV BMLF1-specific clonotype protein sequence identified by MIRA in a library of 10^9^ simulated sequences generated by VDJ recombination. The relative complexity of each clonotype was measured as the abundance of each sequence in the library of 10^9^ simulated clonotypes with more abundant clonotypes being easier to generate than those that are less abundant. **B)** Plot of the frequencies of each shared (identified in more than one individual) and not shared Flu M1- and EBV BMLF1-specific clonotype nucleotide sequence identified by MIRA. Blue lines indicate the median observations (A) and frequencies (B) for each group.

Productive T cell responses during an infection, for example, require the presence of antigen-specific T cell clonotypes prior to clonal expansion. We reasoned that sequences that are most likely to be shared are simpler to generate via VDJ recombination and have higher avidity thus maximizing the likelihood for clonal expansion when cognate antigen is present. To assess the sequence complexity of shared antigen-specific clonotypes we created a library of 10^9^ simulated clonotypes generated by VDJ recombination [33]. In this library simpler sequences were more readily generated and thus more abundant than relatively complex clonotype sequences. We then focused on the two antigens that yielded abundant antigen-specific clonotypes by MIRA in all 5 individuals tested and asked whether shared clonotypes (identified in at least two individuals) were easier to generate than those sequences identified in only one individual. Shared clonotypes specific to Flu M1 and EBV BMLF1 are more abundant in the library and therefore more simple to generate than sequences restricted to a single individual (**Fig 5A**; p-values 0.005 and 0.005, respectively).

Multiple factors determine the frequency of a clonotype. On average higher avidity sequences are likely to dominate and reach higher frequency after clonal expansion. We therefore assessed the frequency of shared and non-shared clonotypes determined to be specific to Flu M1 and EBV BMLF1 by MIRA. The median frequencies of the shared and non-shared EBV BMLF1-specific clonotypes are 7.6x10^-5^ and 3.2x10^-5^, respectively (p-value 0.034), and shared and non-shared Flu M1-specific clonotypes are 2.1x10^-5^ and 6.5x10^-6^, respectively (p-value 0.0045) (**Fig 5B**).

### Fraction of shared clonotypes with different antigen specificities

Multiple nucleotide sequences can encode the same amino acid sequence. We noted that a substantial number of shared Flu M1-specific protein sequences were encoded by more than one nucleotide sequence (**Table 1**). In a single donor, for example, one amino acid sequence was encoded by 5 distinct nucleotide sequences.

We wanted to determine whether the extent of clonotype protein sequence sharing between individuals differed based on antigen specificity so we calculated the fraction of shared clonotypes between all possible pairs of individuals. Since we demonstrated above that a clonotype identified to be specific to a test antigen in an index individual can be predicted to have the same specificity in another individual we included in the shared category any matching clonotype enriched in at least one of the expected antigen address pools. We noted a substantially increased fraction of shared Flu M1-specific clonotype sequences compared to the others tested (p-values for Flu M1 versus CMV pp65, EBV BMLF, EBV BRLF, EBV LMP2 are 0.0002, <0.0001, <0.0001 and <0.0001, respectively; **S6 Fig**).

### Dynamics of clonotypes over time

**Fig 6:**
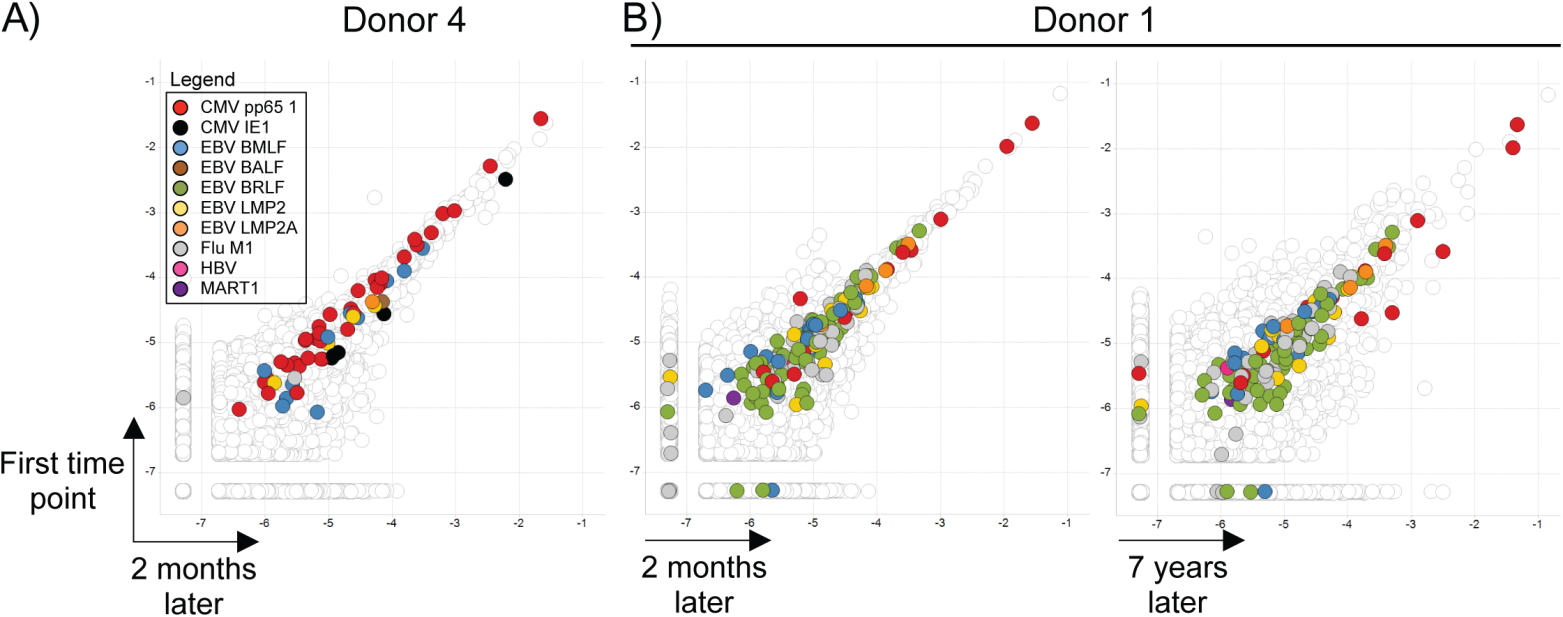
Frequency (Log_10_) dynamics of antigen-specific clonotypes. **A)** Plot of all antigen-specific clonotypes identified from donor 4 with dextramers between time points spaced two months apart. **B)** Plot of all antigen-specific clonotypes identified from donor 1 between time points spaced two months (left) and seven years apart (right). Antigen-specific clonotypes are indicated with colored dots (see legend) and were identified from cells from the first time point of each donor.

Once identified, antigen-specific clonotypes can be identified in other samples from the same individual without the MIRA assay by repertoire sequencing alone. From one individual we obtained and sequenced an additional PBMC sample collected 2 months after the sample used to identify antigen-specific clonotypes and plotted the antigen-specific clonotype frequencies at each time point (**Fig 6A**). We also plotted the frequency dynamics of the antigen-specific TCRb clonotypes identified at the first time point from another individual over a 2 month (**Fig 6B;** left) and 7 year interval (**Fig 6B**; right). The antigen-specific clonotype frequencies are remarkably stable even over a long period of time. Of all the antigens, we noted that a subset of the CMV pp65-specific clonotypes increased substantially in frequency between the 2 samples collected 7 years apart (**S7 Fig**).

## Discussion

Immune receptor repertoire sequencing is a powerful method to enumerate and quantitate distinct clonotypes at different time points or tissues in a sensitive manner. Unfortunately antigens recognized by individual TCRs cannot be readily identified from the sequence. We have demonstrated the development of MIRA by combining repertoire sequencing with immune assays to identify antigen-specific clonotypes for 30 antigens simultaneously. Hundreds of clonotypes were identified at frequencies as low as 1 per million T cells.

This assay provides three important readouts: 1) the sequence of every clonotype specific for a given set of antigens in a sample, 2) the number and frequency of each clonotype specific to a particular antigen, and 3) the sum frequency of the clonotypes specific for each antigen. The latter information is equivalent to other measures of antigen specificity including ELISPOT and flow cytometry-based assays with multimers or cytokines. MIRA has multiple advantages over standard immune monitoring techniques. First, given the capability of very deep sequencing and the specificity of the TCR sequences the technology has high sensitivity (10^−6^ or better). However we do note that MIRA detects each antigen-specific T cell clonotype independently. Therefore an immune response involving a large number of very low frequency antigen-specific clonotypes may not be resolved with MIRA if the input number of cells results in a limit of detection that is higher than the frequency of the individual clonotypes. Second, the number and diversity of the clonotypes specific to a particular antigen can be elucidated. Third, the sequence information provides a permanent record of antigen specificity for that clonotype within a particular individual. Once identified, antigen-specific clonotype sequences can be used to query TCR clonotypes obtained from additional serial samples or material from other tissues from the same individual with sequencing alone and without a live cell requirement. Moreover, identifying the same or highly similar clonotype sequences from the same or even a different individual may predict the specificity of these clonotypes without the need for an immune assay. Fourth, many antigen specificities can be assessed concurrently. We demonstrated the use of 30 antigens simultaneously and the approach can be readily extended to hundreds or thousands of antigens. In fact with scale-up from a single to 30 antigens, neither the sensitivity nor the degree of enrichment of the clonotypes identified in the sorted antigen-specific compared to sorted not antigen-specific populations deteriorated. This was directly shown by the high correlation of the identified antigen-specific clonotypes in one of the donor samples assessed with MIRA that was previously published using a single antigen [17].

MIRA allowed us to investigate aspects of clonotypes that were shared between individuals. We demonstrated that these clonotypes, compared to non-shared clonotypes, are simpler and easier to generate via VDJ recombination. In addition they are present at higher frequency which is consistent with them having higher avidity. We observed that the Flu peptide antigen tested elicited a higher proportion of shared clonotypes than those specific to CMV and EBV peptide antigens. This was shown by a higher fraction of different nucleotide sequences coding for the same amino acid within individuals. Independently, there was a higher fraction of Flu-specific clonotypes shared between individuals compared to those specific to CMV or EBV antigens. The tendency of Flu-responsive clonotypes to be shared between individuals more frequently may be explained by evolutionary selection for simpler VDJ rearrangements that are specific to Flu. This seems consistent with the data where all Flu-specific clonotype sequences seem to be simpler than clonotypes specific to EBV BMLF1, regardless of whether they are shared or not between individuals. In addition the nature of repeated (seasonal) Flu infections might contribute to the higher fraction of sharing. Multiple cycles of clonotype expansion leading to repeated selection of higher avidity clonotypes may increase the likelihood of shared clonotypes between individuals as well as the number of clonotypes with the same amino acid within an individual.

Among the tested antigens, once a TCRb clonotype was deemed antigen-specific in one individual then the likelihood it had the same specificity in another individual was very high (92%). One may conclude that TCRa is not relevant for antigen specificity. More likely this conclusion is biased by the fact that we require the clonotype to be detected in another donor. In these experiments we are assessing relatively high frequency (>10^−6^) TCRb clonotypes that are the result of clonal expansion (*i*.*e*., memory T cells). Out of multiple T cells with the same TCRb sequence, those containing a TCRa with specificity to one of the tested viral antigens are more likely to expand than T cells with a TCRa that confers a different antigen specificity. Therefore it is likely that the conclusion may be less dramatic with a set of different antigens, especially upon assessment of lower frequency clonotypes. Nevertheless the ability to predict antigen specificity based on matching of a clonotype sequence to one with known antigen specificity enhances the power of the technology. A database of clonotypes specific to a set of antigens from a set of individuals using MIRA can allow the identification of antigen-specific clonotypes in another set of individuals by simply querying sequences compiled in the database. This type of database would be very useful in determining a large fraction of clonotypes specific to an antigen of interest. Extending MIRA to hundreds or thousands of antigens enhances the value of the database. The fraction of clonotypes whose antigen specificity can be discerned increases with the size of the database. Using a model that utilizes sequences with the distribution of “complexity” seen in human TCRb repertoires, a database of 10^8^ independent CDR3 amino acid sequences can be obtained from 10^9^ independently generated sequences. Applying MIRA to about 10,000 samples can generate a database of this magnitude. We estimate about 50% of the clonotypes derived from a sample from a new individual not yet in the database would be contained in the database (data not shown). For viral antigens we have demonstrated that a clonotype with a determined antigen specificity by MIRA in one individual can be predicted to have the same specificity when detected in another individual. The degree this can be readily extended to other antigens is still unknown. TCRa information can be easily added to the database in order to improve the antigen specificity prediction.

The multiplex approach we describe is not limited to CD8 T cells and can be easily adapted to assess antigen-specific CD4 T cells. In this work we focused on identification of antigen-specific CD8 T cells by using dextramer binding or expression of an activation marker (CD137) following peptide stimulation. In an analogous fashion we have demonstrated the identification of antigen-specific CD4 T cells by using peptide stimulation and expression of a different activation marker (data not shown) and the assay worked as robustly as for CD8 cells.

In conclusion application of MIRA to identify antigen-specific clonotypes using hundreds or thousands of antigens would allow the elucidation of TCR sequence, frequency, and antigen specificity providing an unprecedented breadth and depth in studying immune responses. The study of large numbers of samples using MIRA and the dynamics of these clonotypes in other samples from the same individuals can potentially make a profound improvement in our understanding of immune responses as well as accelerate biomarker discovery and therapeutics development in multiple health related states like infection, autoimmune disease and cancer.

## Materials and Methods

### Samples and reagents

Cryopreserved and characterized PBMCs were purchased from Cellular Technology Limited (CTL; Shaker Heights, OH). IFN-γ ELISPOT analyses with a subset of peptide antigens were performed independently by Cellular Technology Limited (Shaker Heights, OH). MHC Class I HLA-A*02-restricted dextramers (Immudex) were used according to manufacturer’s instructions. Prior to mixing different combinations of individual dextramers, D-biotin (Life Technologies) was added to the tube, according to manufacturer’s instructions. Fluorescently conjugated antibodies (BioLegend) to the following cell surface markers were used: CD3 (SK7), CD4 (OKT4) CD8 (SK1) and CD137 (4-1BB). Peptides were synthesized by CPC Scientific (Sunnyvale, CA). Mixtures of reconstituted peptides were prepared by adding an equal volume of each peptide to the mixture (note the final concentration of each peptide during stimulation was 1ug/ml).

### Antigen-Specific T Cell Assays, Flow Cytometry and Cell Sorting

Antigen-specific T cells were identified using one of two approaches: 1) dextramer binding or 2) CD137 upregulation following overnight incubation with mixtures of peptides. Dextramer-specific T cells were identified by incubating PBMCs with pools of 8 dextramers according to manufacturer’s instructions. We obtained antigen-specific T cells via CD137 upregulation following brief in vitro incubation as outlined previously [17]. Complete media containing 15% Fetal Bovine Serum (FBS), non-essential amino acids, glutamine and antibiotics was used for peptide incubations. Thawed PBMCs were washed and plated at ~400,000 cells per well, in replicates in complete media (in 96-well U-bottom plates). Unconjugated antibodies directed against CD28 and CD49d were then added to the wells containing the suspended cells. Pools of peptides derived from 30 different viral/tumor-associated antigens were added directly to the cell/antibody mixture. Following addition of peptides, cells were incubated at 37°C for ~18 hours. At the end of the incubation, replicate wells of cells were harvested from the culture and pooled and then stained with antibodies for analysis and sorting by flow cytometry. Cells were then washed and suspended in PBS containing FBS (2%), 1mM EDTA and 4′,6-diamidino-2-phenylindole (DAPI) for exclusion of non-viable cells. Cells were acquired and sorted using a FACSAria (BD Biosciences) instrument. Sorted antigen-specific (CD4-CD8+dextramer+ or CD3+CD8+CD137+) and not antigen-specific (CD4-CD8+dextramer- or CD3+CD8+CD137-) T cells were pelleted and lysed in RLT Plus buffer for nucleic acid isolation. Analysis of flow cytometry data files was performed using FlowJo (Ashland, OR).

### Nucleic acid to clonotype determination

RNA and DNA was isolated using AllPrep DNA/RNA mini and/or micro kits, according to manufacturer’s instructions (Qiagen). RNA was reverse transcribed to cDNA using Vilo kits (Life Technologies). TCRβ amplification, sequencing and clonotype determination are described in detail elsewhere [17]. All dextramer-based MIRA experiments used DNA as input material for sequencing. All peptide-based MIRA experiments used RNA as input material for sequencing.

### Antigen-Specific Clonotype Selection Criteria

Antigen-specific and not antigen-specific T cells were sorted from 8 or 10 aliquots, depending on the assay (dextramer- or peptide-based, respectively). Enriched clonotypes were identified as those clonotypes present at a greater than 20-fold higher frequency in the sorted antigen-specific T cell population compared to the sorted not antigen-specific population from the same aliquot. If a clonotype was present in the sorted antigen-specific population but absent from the sorted not antigen-specific population from a particular pool then the fold frequency change could not be calculated. In these cases if a clonotype was present only in the sorted antigen-specific population (and absent from the sorted not antigen-specific) then it was considered enriched, regardless of the frequency. Clonotypes were deemed antigen-specific if, based on their enrichment in 4 of 8 (dextramers) or 5 of 10 (peptides) pools, they matched an individual dextramer or peptide address. Clonotype protein CDR3s were defined as those sequences flanked by C and F within the full clonotype protein sequences.

### Clonotype sequence complexity

To determine clonotype uniqueness we simulated the generation of 10^9^ clonotypes [33]. These clonotypes were generated based on a model that include parameters of the likelihood of having specific V, D, and J segments, as well as V, D, and J deletion sizes and N base insertions. These factors were obtained from a large database of clonotypes. The simulation then generated 10^9^ clonotypes using the probability parameters of the model. The complexity measure for a clonotype was simply the number of exact matches in the library of the 10^9^ clonotypes.

### Statistical analysis

Non-parametric Mann-Whitney tests were used to calculate p-values in Fig 5, S5 Fig and S6 Fig.

## Supporting Information

S1 Fig Overview of a singleplex assay. Assay procedure outline: 1) Incubate cells with immune assay reagent (dextramers, peptides, etc.), 2) Sort into two T cell populations: antigen-specific and not antigen-specific, 3) Sequence TCR, 4) Identify antigen-specific TCR clonotypes as those at higher frequency in the sorted antigen-specific population compared to the population that is not antigen-specific.
(TIF)

S2 Fig MIRA results are highly reproducible. Plot shows number (bars) and sum frequency (red circles) of antigen-specific clonotypes identified by MIRA from replicate 2 (‘month 2’) from donor 1. For comparison, replicate 1 from this donor is shown in Fig 2 and S1 Table.
(TIF)

S3 Fig Identification of antigen-specific clonotypes from an earlier time point from the same individual. Plot shows number (bars) and sum frequency (red circles) of ‘month 0’ antigen-specific clonotypes identified by MIRA from PBMCs collected from blood drawn 2 months prior to ‘month 2’ results from donor 1 shown in Fig 2, S2 Fig and S2 Table.
(TIF)

S4 Fig Peptide-based MIRA set-up. The PBMC sample is divided into an equal number of aliquots (A to J, indicated in red at top) matching the total number of peptide, or antigen pools. Each peptide is assigned to a unique subset, or “address”, of exactly 5 of 10 pools as indicated in the right column. Individual peptide assignments are indicated with an “X”. The CMV IE1 peptide, for example, was assigned to subsets B, D, E, F and J but not A, C, G, H or I.
(TIF)

S5 Fig Presence of antigen-specific clonotype protein sequences in other individuals is HLA-A*02-dependent. All antigen-specific T cell clonotype protein sequences identified with dextramers and peptides were used to query the T cell repertoires from an independent set of HLA-A*02-positive (n = 7) and HLA-A*02-negative (n = 6) individuals. The number of clonotypes identified in each donor from each group that matches a query sequence is shown in the plot. Horizontal lines indicate mean and SEM.
(TIF)

S6 Fig The fraction of clonotype protein sequences shared between individuals varies depending on antigen-specificity. Flu M1-, CMV pp65-, EBV BMLF-, EBV BRLF- and EBV LMP2-specific clonotypes identified from each individual with the peptide-based MIRA assay were queried in each of the other 4 individuals. All possible pairs were assessed from each of the 5 donors and the fraction of clonotypes identified in one individual and found in another individual was plotted. Horizontal lines indicate mean and SEM.
(TIF)

S7 Fig Frequencies of antigen-specific clonotypes plotted over a 7 year period. CMV pp65-, Flu M1- and EBV BRLF1-specific clonotypes were identified at the 0 month time point from donor 1 and sum frequencies were plotted at all time points.
(TIF)

S1 Table Number and sum frequency of antigen-specific clonotypes identified in each donor with dextramer-based MIRA. (TIF)

S2 Table Number and sum frequency of antigen-specific clonotypes identified by MIRA from a replicate PBMC sample from donor 1. For comparison, results from the first replicate from this donor are shown in Fig 2 and S1 Table.
(TIF)

S3 Table Identification of the same antigen-specific clonotypes from an earlier time point from the same individual. Table shows number and sum frequency of ‘month 0’ antigen-specific clonotypes identified by MIRA from PBMCs collected from blood drawn 2 months prior to samples from donor 1 used to generate data shown in Fig 2, S2 Fig and S2 Table.
(TIF)

S4 Table Mixing PBMCs from different donors. The number of antigen-specific clonotypes identified by dextramer-based MIRA from a mixed sample containing PBMCs from 3 donors (donors 1, 2 and 4).
(TIF)

S5 Table RNA input improves clonotype detection. Table shows the number of antigen-specific clonotypes identified by dextramer-based MIRA from a replicate experiment using a mixed sample containing PBMCs from 3 donors (donors 1, 2 and 4). Note these results are from a replicate of the experiment outlined in S4 Table. The two columns at right indicate the results from either DNA or RNA isolated from the same populations of sorted antigen-specific and not antigen-specific cells from each of the 8 aliquots as outlined in Fig 1B.
(TIF)

S6 Table Number and frequency of antigen-specific clonotypes identified with peptide-based MIRA. (TIF)

S7 Table Antigen-specific clonotype protein sequences identified with peptide-based MIRA assay that differ by one amino acid (shown in red). 15 clusters of antigen-specific clonotype protein sequences are listed with antigen specificity determination indicated. CDR3 sequences are underlined.
(TIF)

S8 Table Prediction of clonotype antigen specificity in individuals based on MIRA antigen assignment in an index individual. The number of clonotypes from an individual matching an antigen-specific query clonotype protein sequence identified in an index individual are indicated in the second column. The observed number of matching clonotypes that were enriched in the positive fraction of at least one of the pools of the expected antigen address of the query sequence are indicated in the third column. The number of matching clonotypes expected to be enriched in the antigen address pools if clonotype enrichment occured randomly are indicated in the fourth column. The computed p-values of observed versus expected events are shown in the last column.
(TIF)
