# Supplementary figures and images for: Multiplex Identification of Antigen-Specific T Cell Receptors Using a Combination of Immune Assays and Immune Receptor Sequencing
Source: PLoS One. 2015 Oct 28;10(10):e0141561. doi: 10.1371/journal.pone.0141561 (PMC4624875; doi:10.1371/journal.pone.0141561)

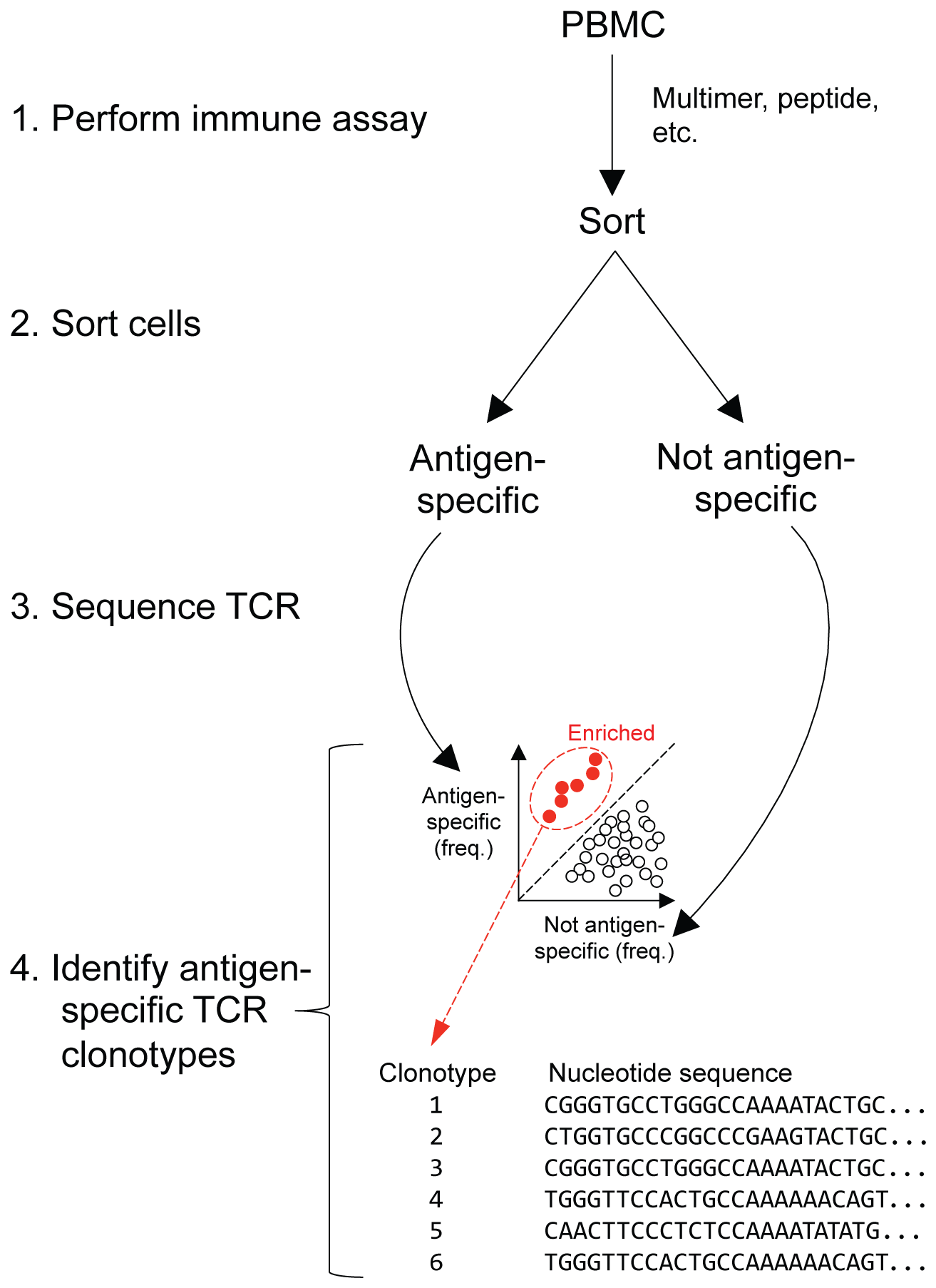

Supplement: S1 Fig — Assay procedure outline: 1) Incubate cells with immune assay reagent (dextramers, peptides, etc.), 2) Sort into two T cell populations: antigen-specific and not antigen-specific, 3) Sequence TCR, 4) Identify antigen-specific TCR clonotypes as those at higher frequency in the sorted antigen-specific population compared to the population that is not antigen-specific. (TIF) [file pone.0141561.s001.tif]

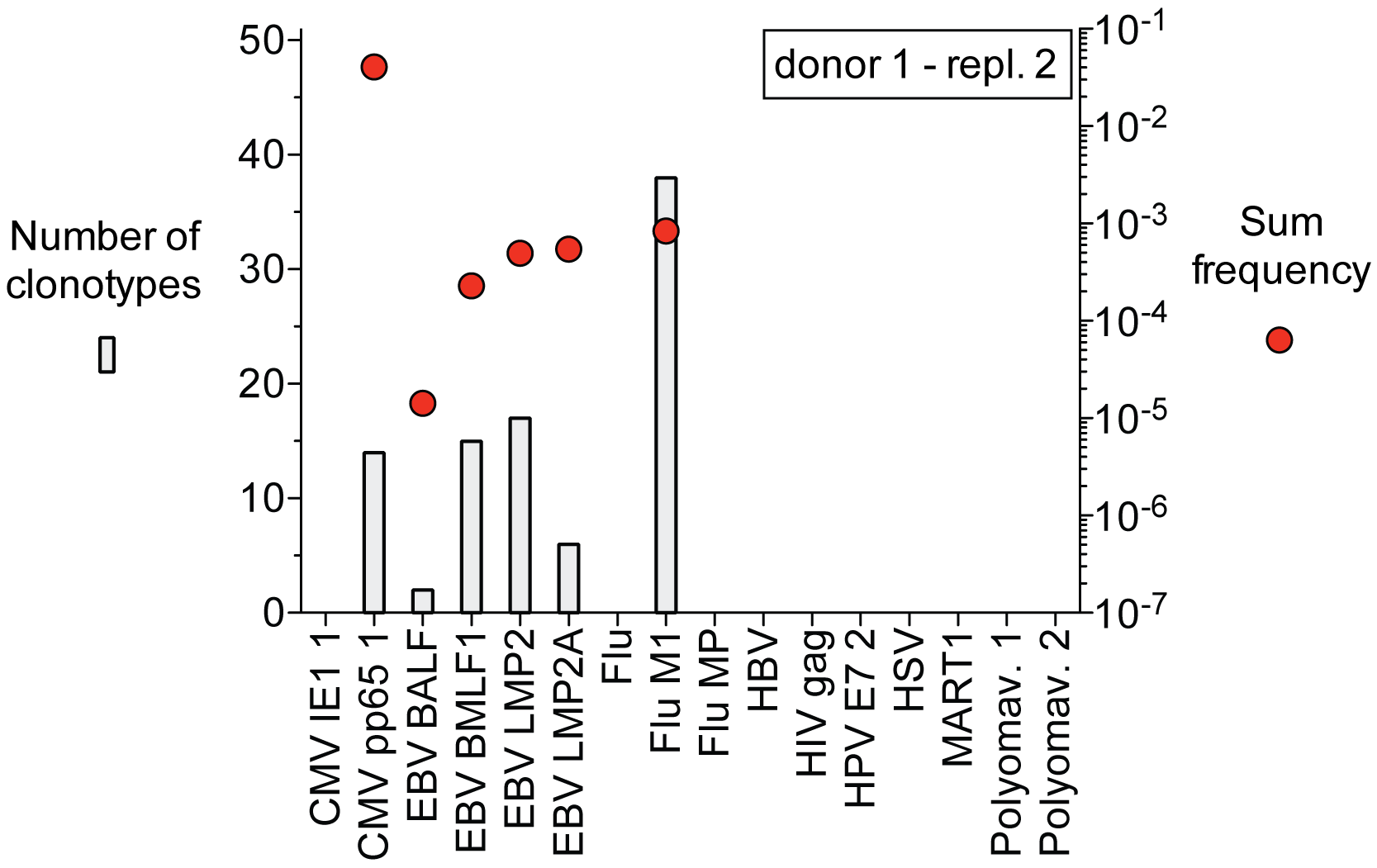

Supplement: S2 Fig — Plot shows number (bars) and sum frequency (red circles) of antigen-specific clonotypes identified by MIRA from replicate 2 (‘month 2’) from donor 1. For comparison, replicate 1 from this donor is shown in Fig 2 and S1 Table. (TIF) [file pone.0141561.s002.tif]

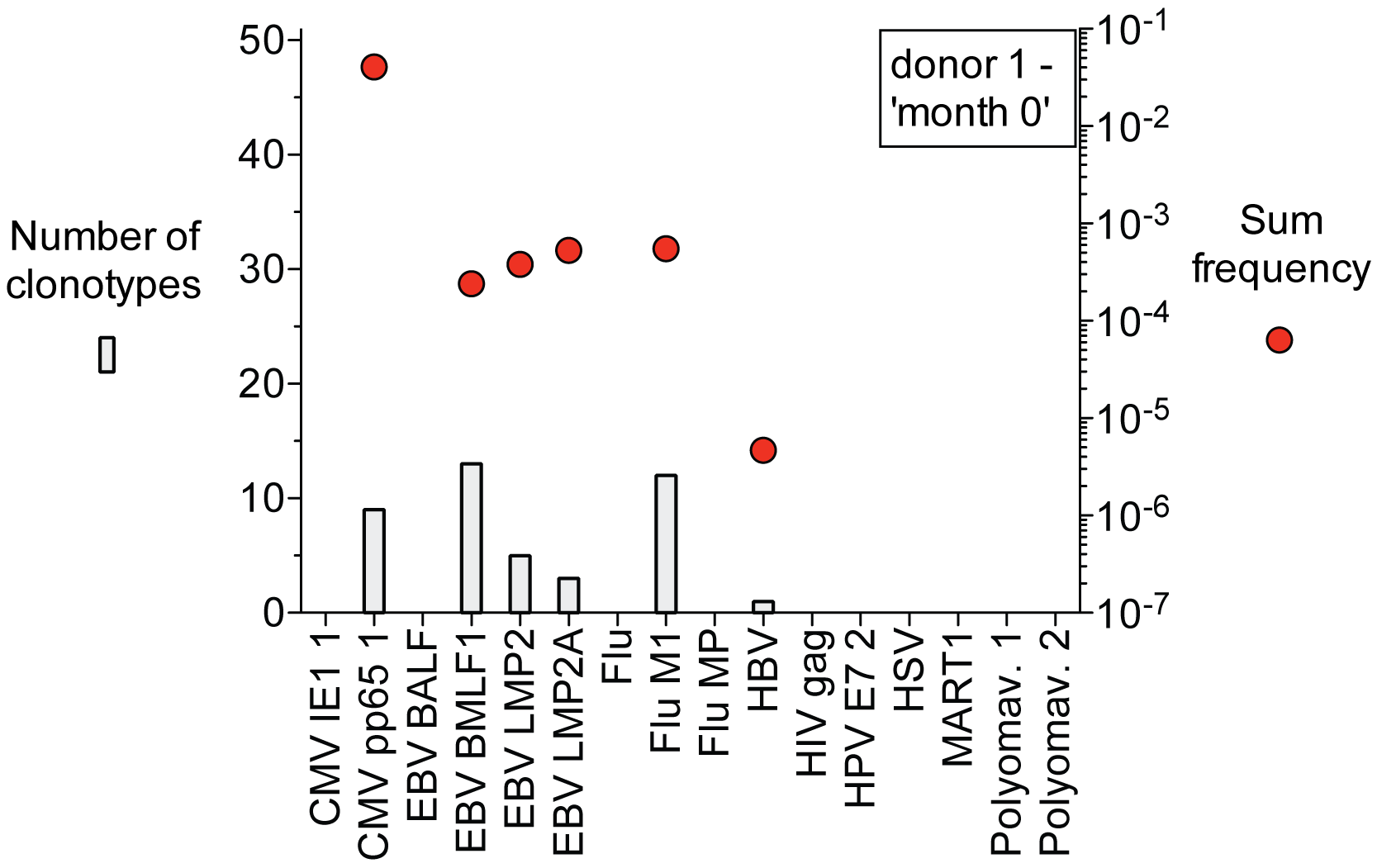

Supplement: S3 Fig — Plot shows number (bars) and sum frequency (red circles) of ‘month 0’ antigen-specific clonotypes identified by MIRA from PBMCs collected from blood drawn 2 months prior to ‘month 2’ results from donor 1 shown in Fig 2, S2 Fig and S2 Table. (TIF) [file pone.0141561.s003.tif]

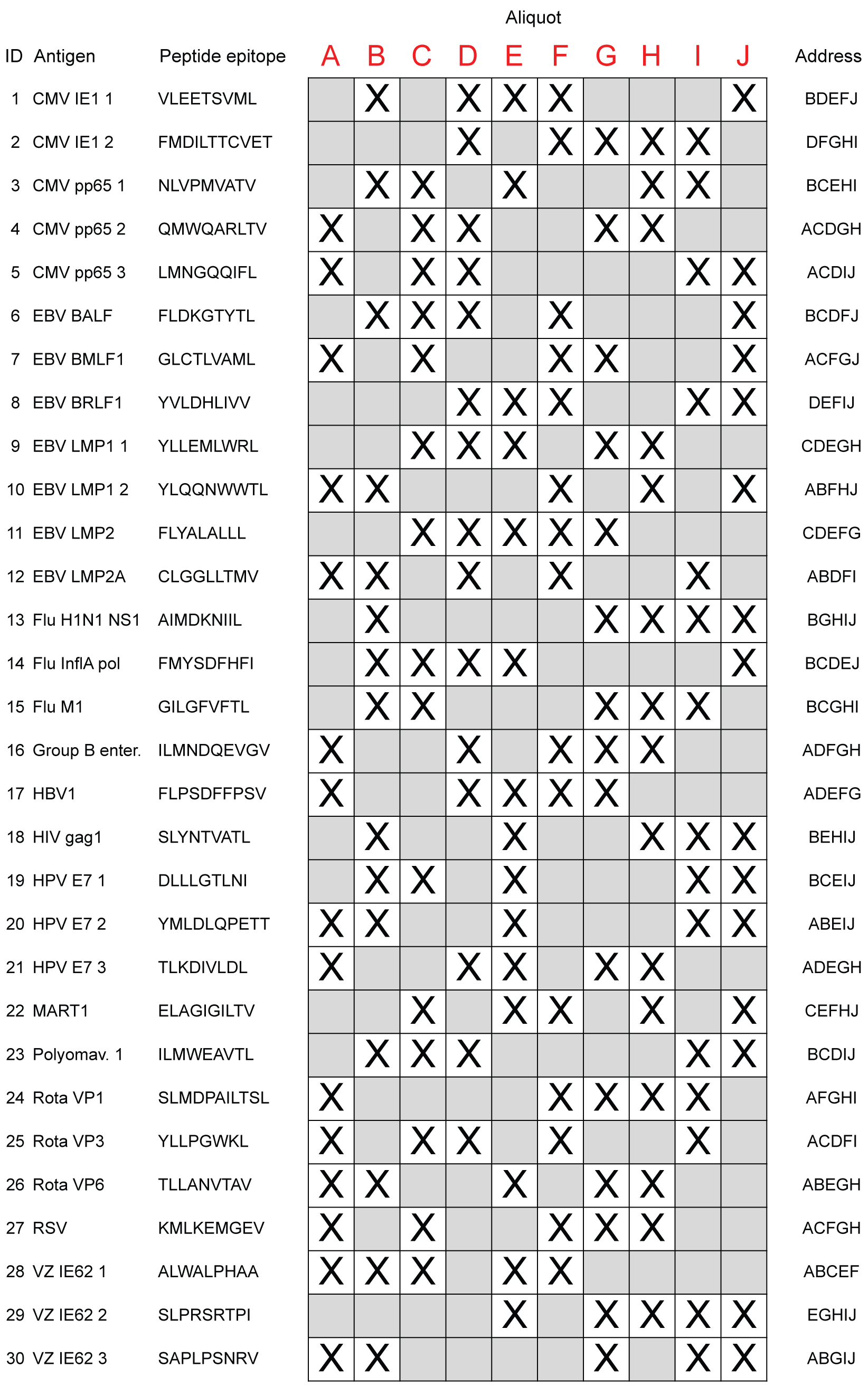

Supplement: S4 Fig — The PBMC sample is divided into an equal number of aliquots (A to J, indicated in red at top) matching the total number of peptide, or antigen pools. Each peptide is assigned to a unique subset, or “address”, of exactly 5 of 10 pools as indicated in the right column. Individual peptide assignments are indicated with an “X”. The CMV IE1 peptide, for example, was assigned to subsets B, D, E, F and J but not A, C, G, H or I. (TIF) [file pone.0141561.s004.tif]

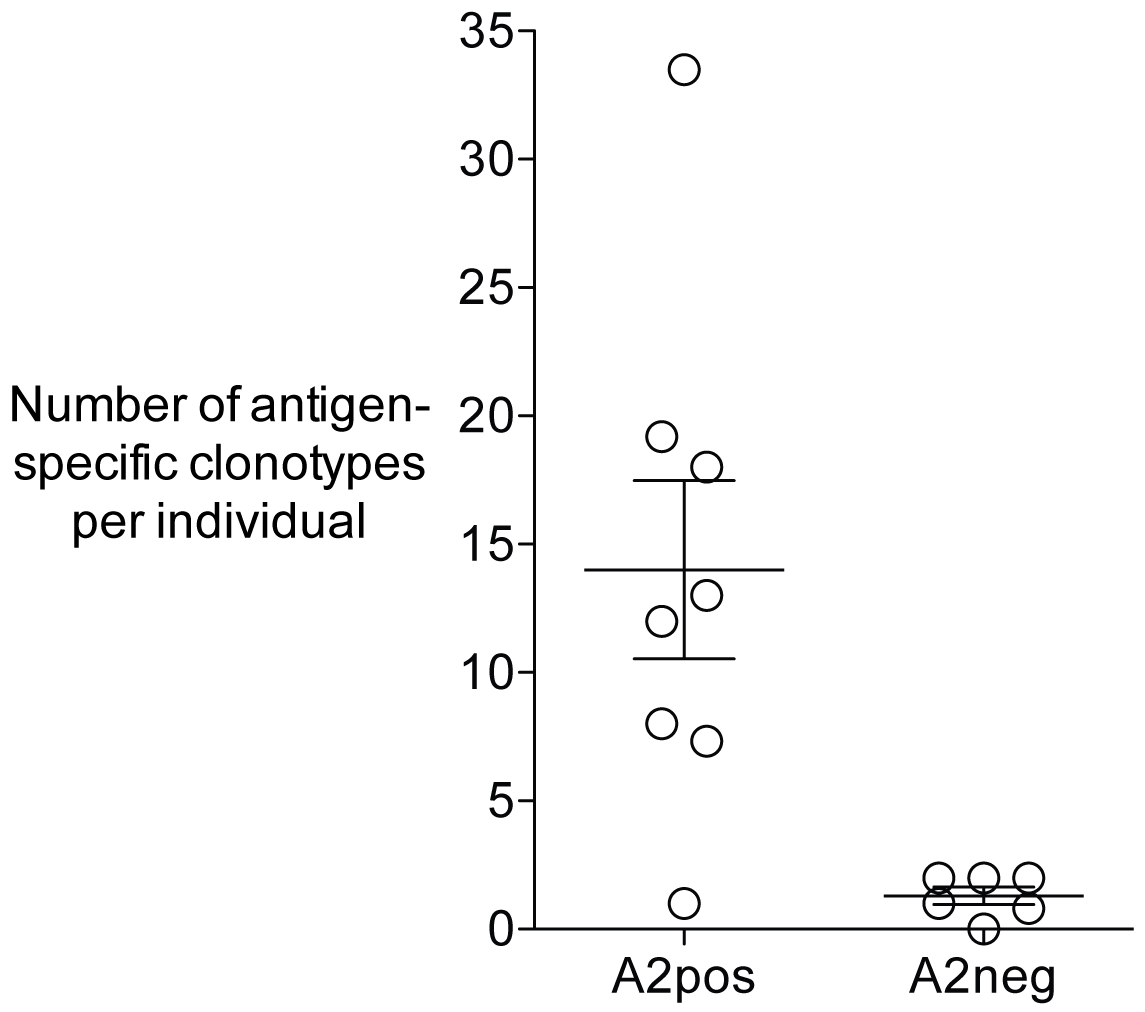

Supplement: S5 Fig — All antigen-specific T cell clonotype protein sequences identified with dextramers and peptides were used to query the T cell repertoires from an independent set of HLA-A*02-positive (n = 7) and HLA-A*02-negative (n = 6) individuals. The number of clonotypes identified in each donor from each group that matches a query sequence is shown in the plot. Horizontal lines indicate mean and SEM. (TIF) [file pone.0141561.s005.tif]

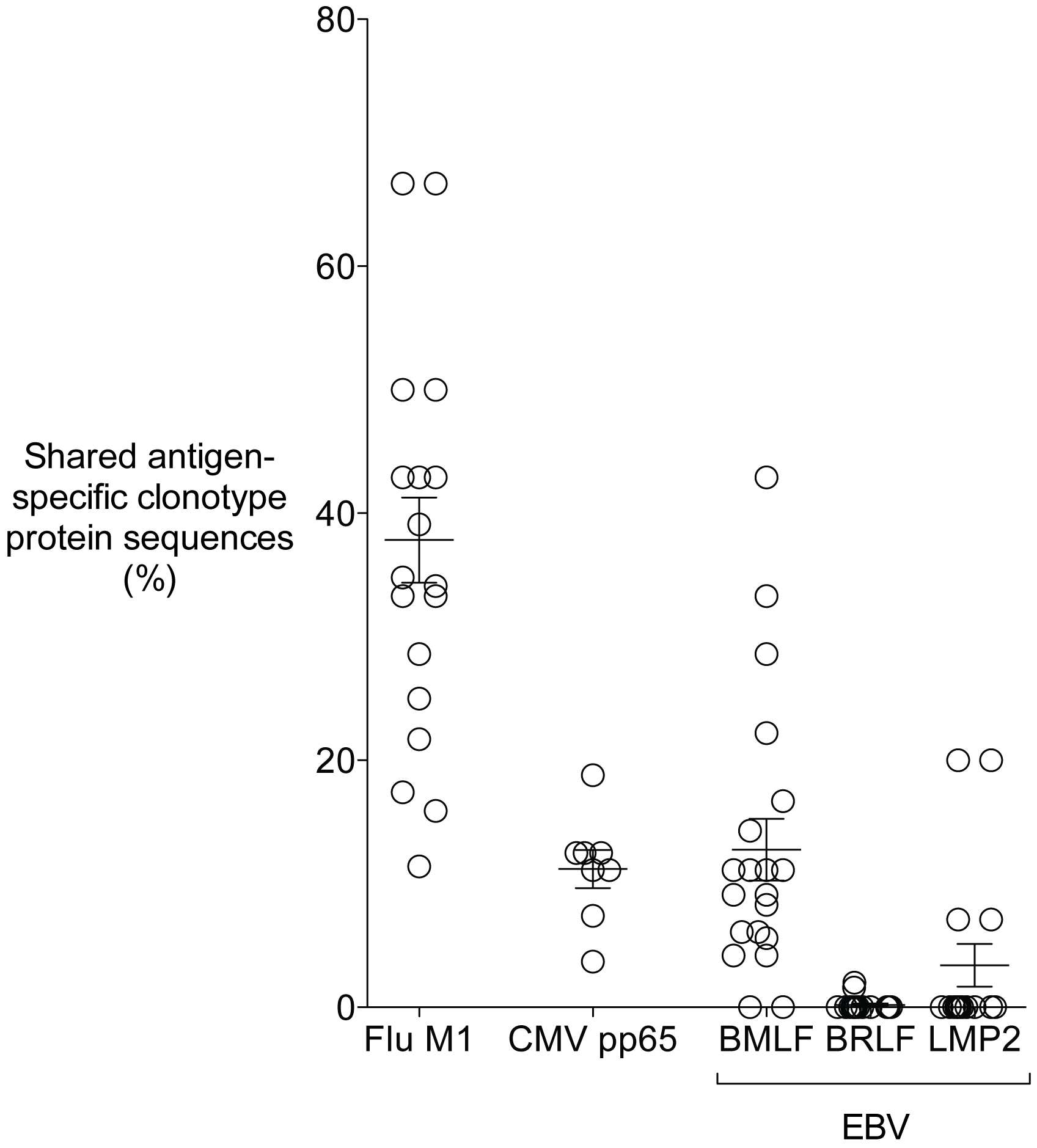

Supplement: S6 Fig — Flu M1-, CMV pp65-, EBV BMLF-, EBV BRLF- and EBV LMP2-specific clonotypes identified from each individual with the peptide-based MIRA assay were queried in each of the other 4 individuals. All possible pairs were assessed from each of the 5 donors and the fraction of clonotypes identified in one individual and found in another individual was plotted. Horizontal lines indicate mean and SEM. (TIF) [file pone.0141561.s006.tif]

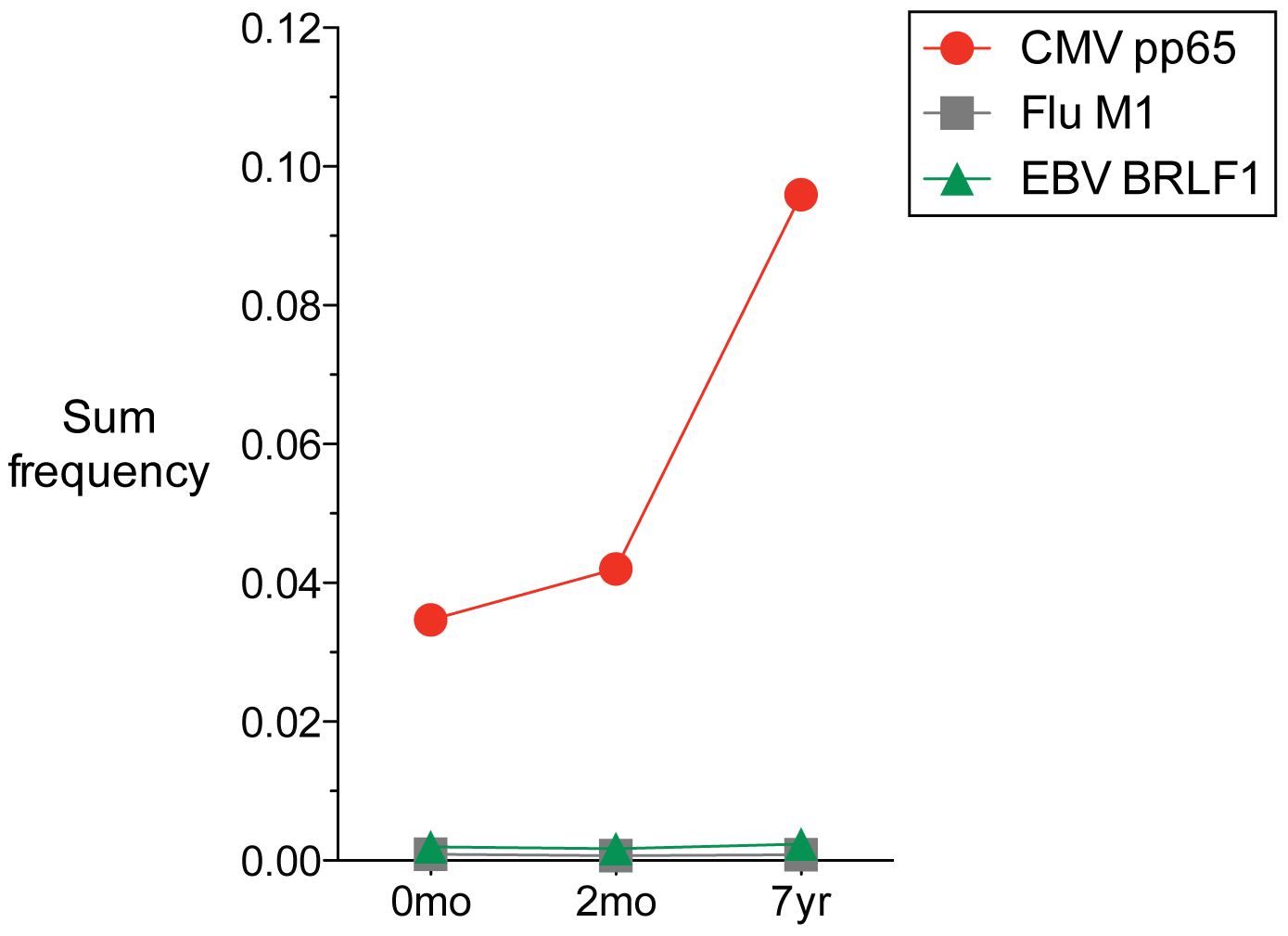

Supplement: S7 Fig — CMV pp65-, Flu M1- and EBV BRLF1-specific clonotypes were identified at the 0 month time point from donor 1 and sum frequencies were plotted at all time points. (TIF) [file pone.0141561.s007.tif]

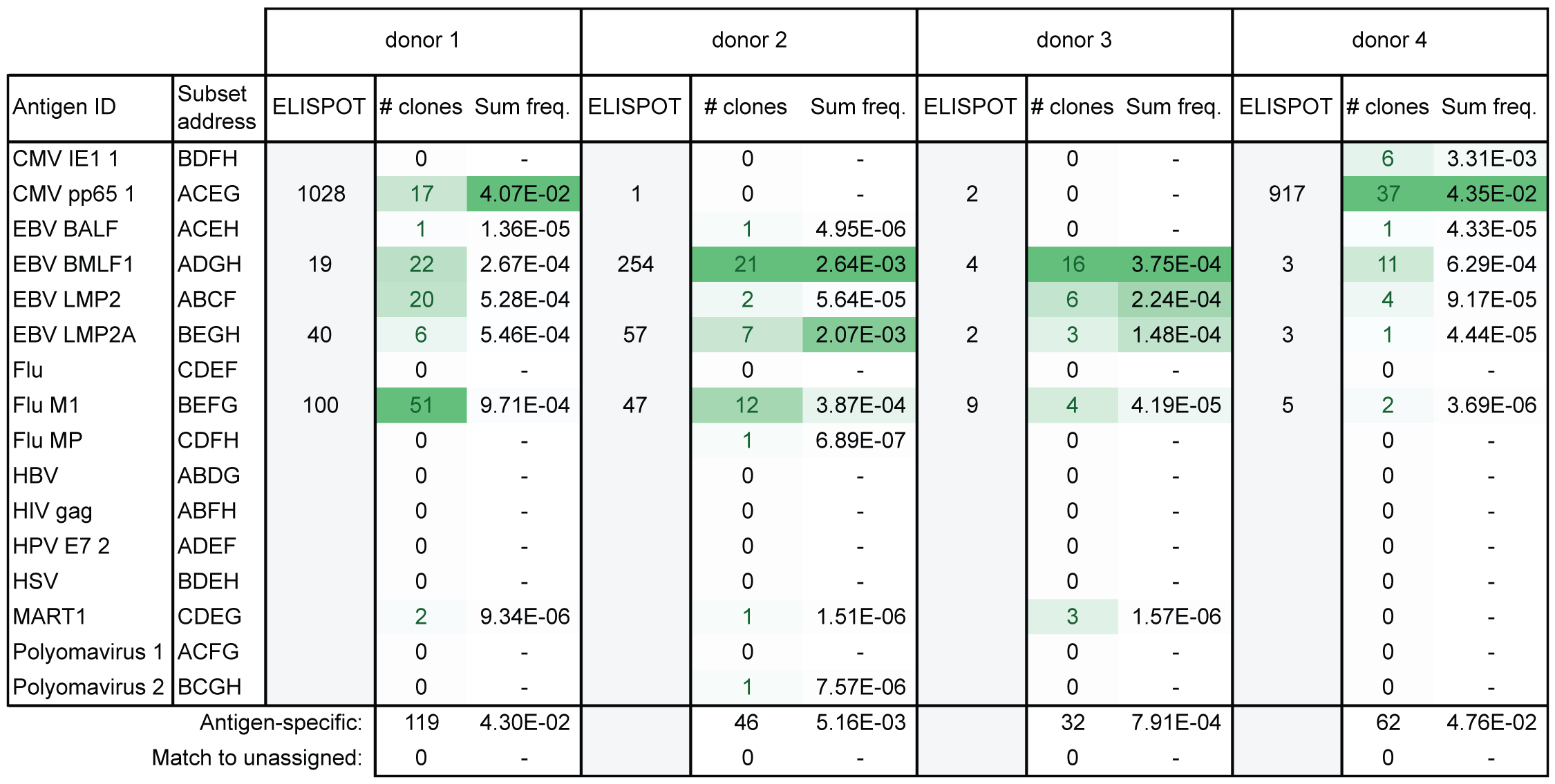

Supplement: S1 Table — (TIF) [file pone.0141561.s008.tif]

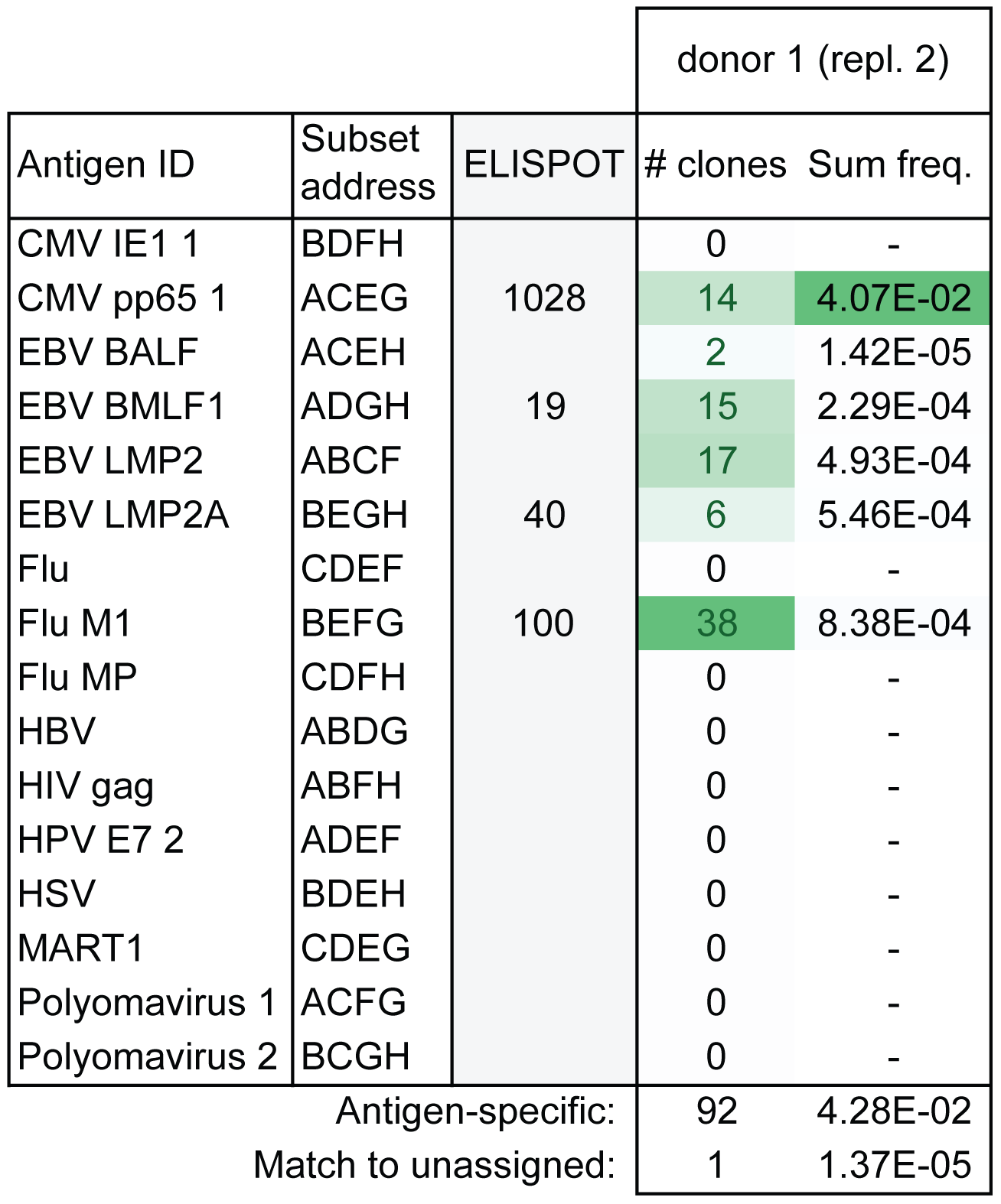

Supplement: S2 Table — For comparison, results from the first replicate from this donor are shown in Fig 2 and S1 Table. (TIF) [file pone.0141561.s009.tif]

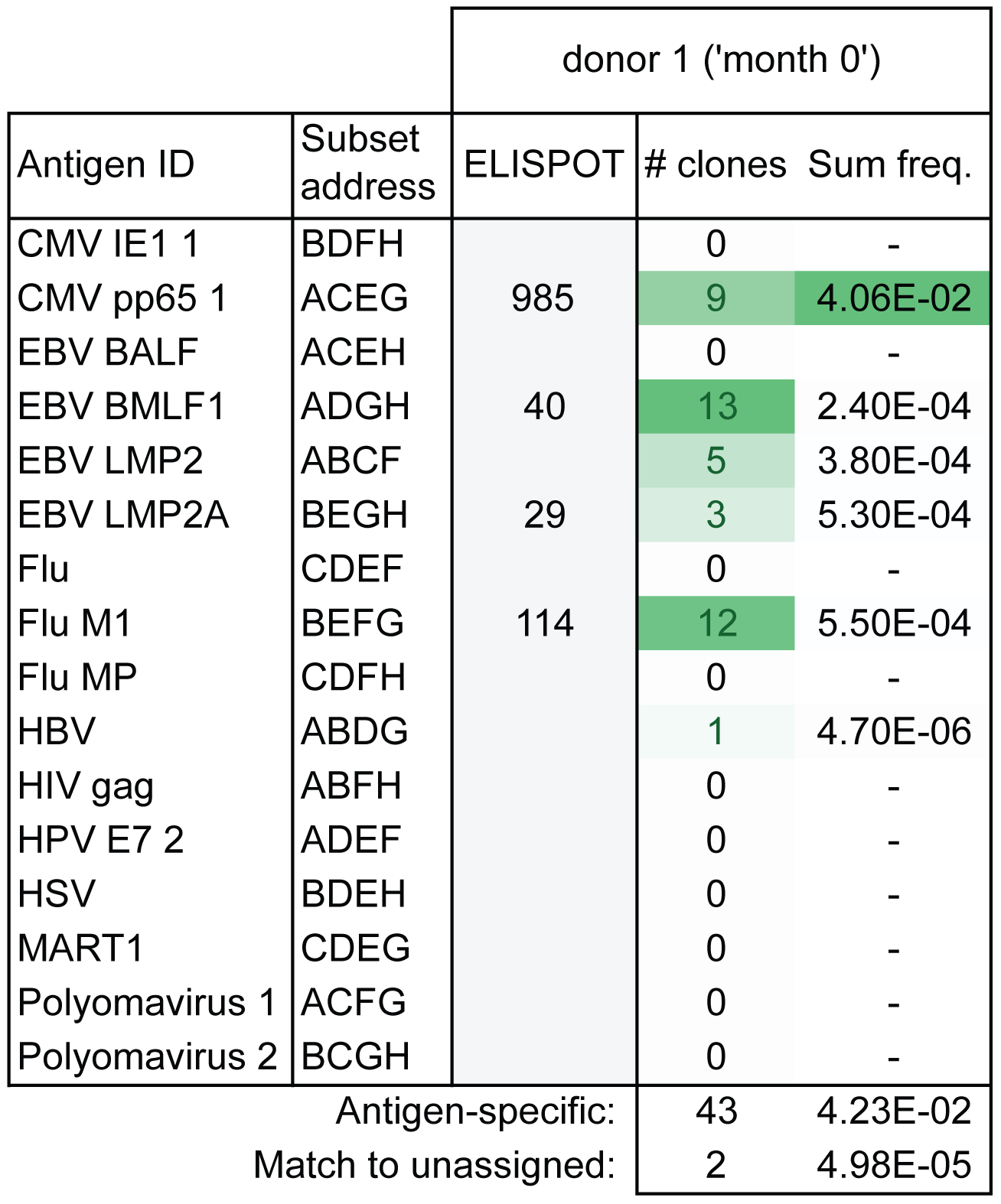

Supplement: S3 Table — Table shows number and sum frequency of ‘month 0’ antigen-specific clonotypes identified by MIRA from PBMCs collected from blood drawn 2 months prior to samples from donor 1 used to generate data shown in Fig 2, S2 Fig and S2 Table. (TIF) [file pone.0141561.s010.tif]

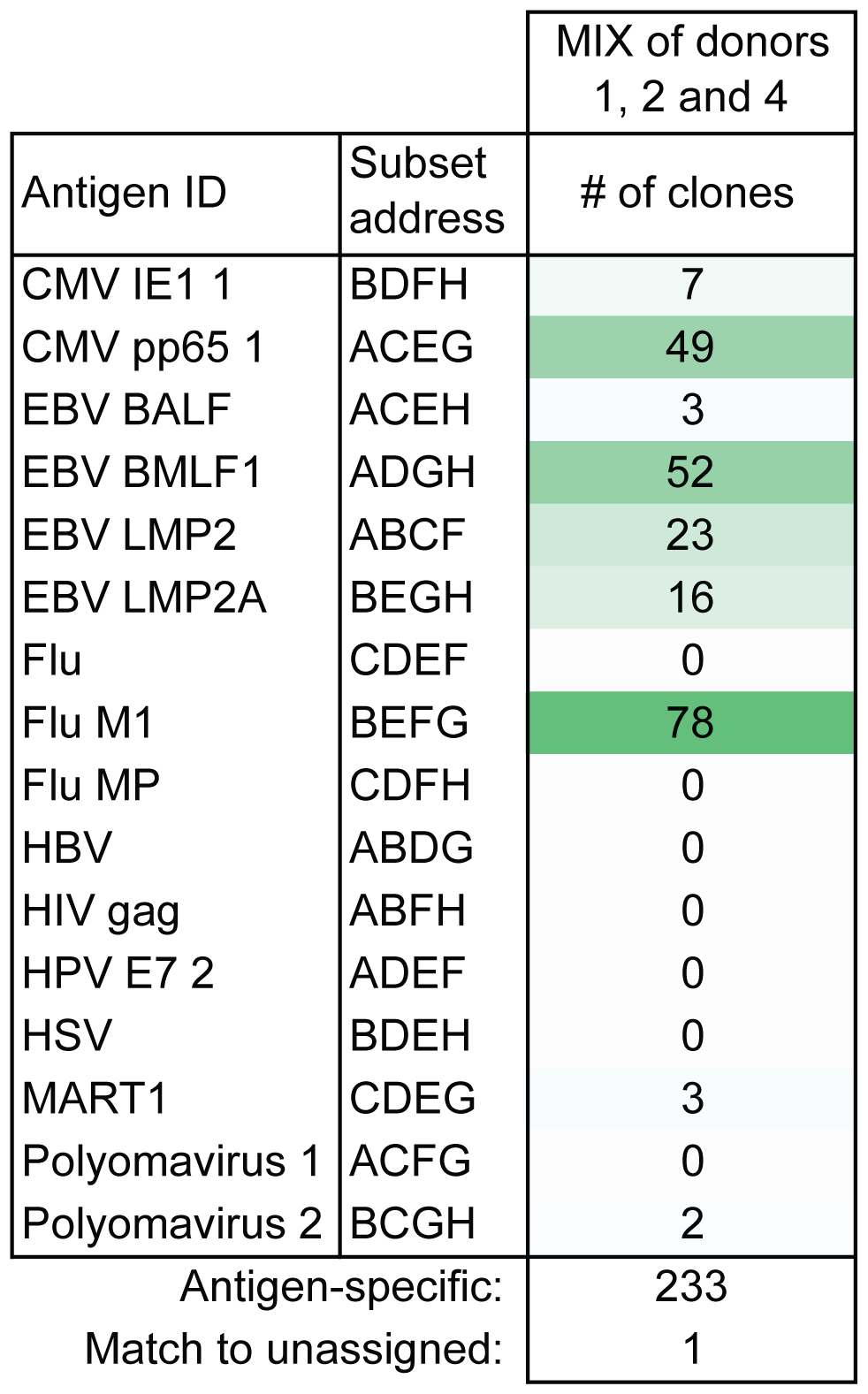

Supplement: S4 Table — The number of antigen-specific clonotypes identified by dextramer-based MIRA from a mixed sample containing PBMCs from 3 donors (donors 1, 2 and 4). (TIF) [file pone.0141561.s011.tif]

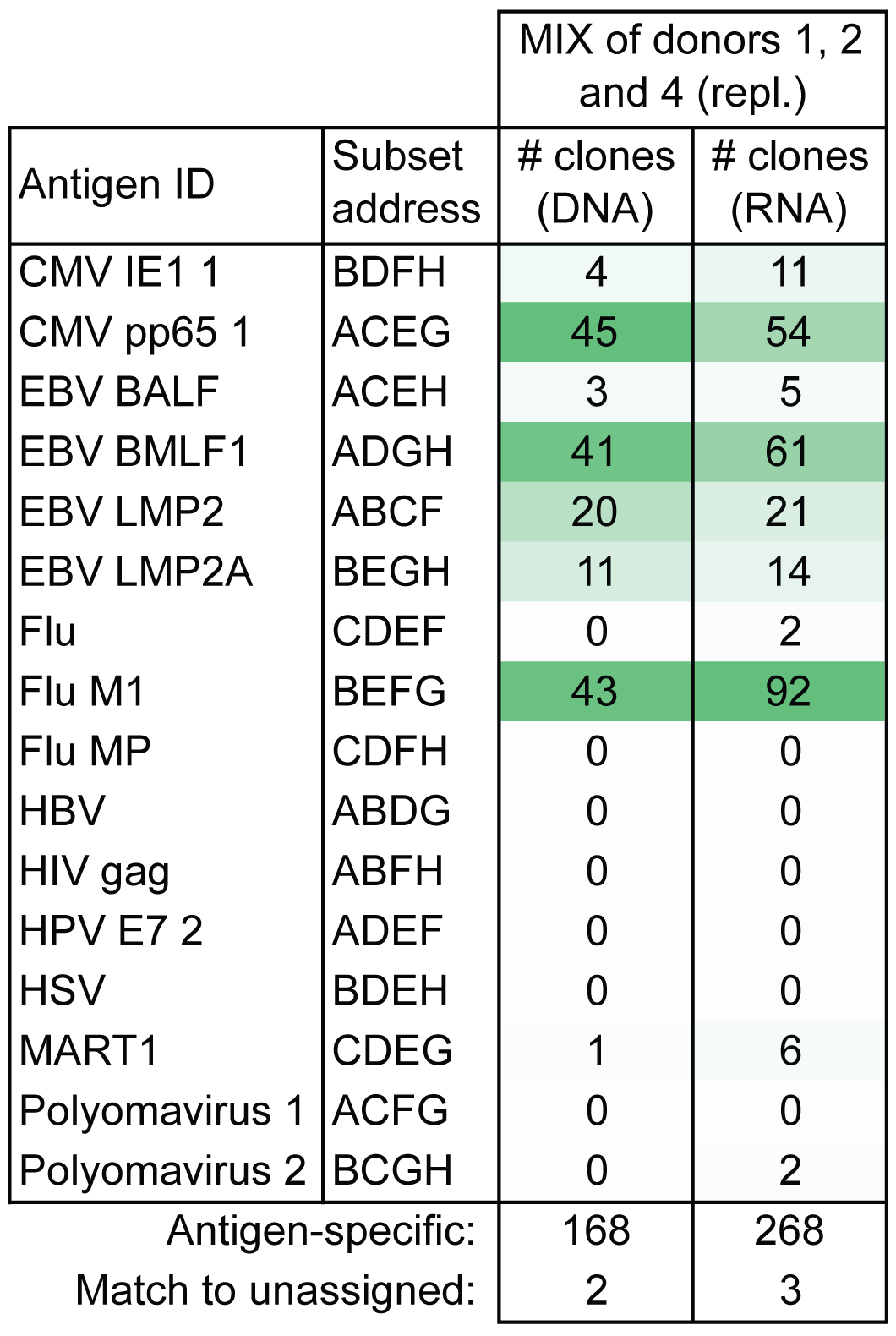

Supplement: S5 Table — Table shows the number of antigen-specific clonotypes identified by dextramer-based MIRA from a replicate experiment using a mixed sample containing PBMCs from 3 donors (donors 1, 2 and 4). Note these results are from a replicate of the experiment outlined in S4 Table. The two columns at right indicate the results from either DNA or RNA isolated from the same populations of sorted antigen-specific and not antigen-specific cells from each of the 8 aliquots as outlined in Fig 1B. (TIF) [file pone.0141561.s012.tif]

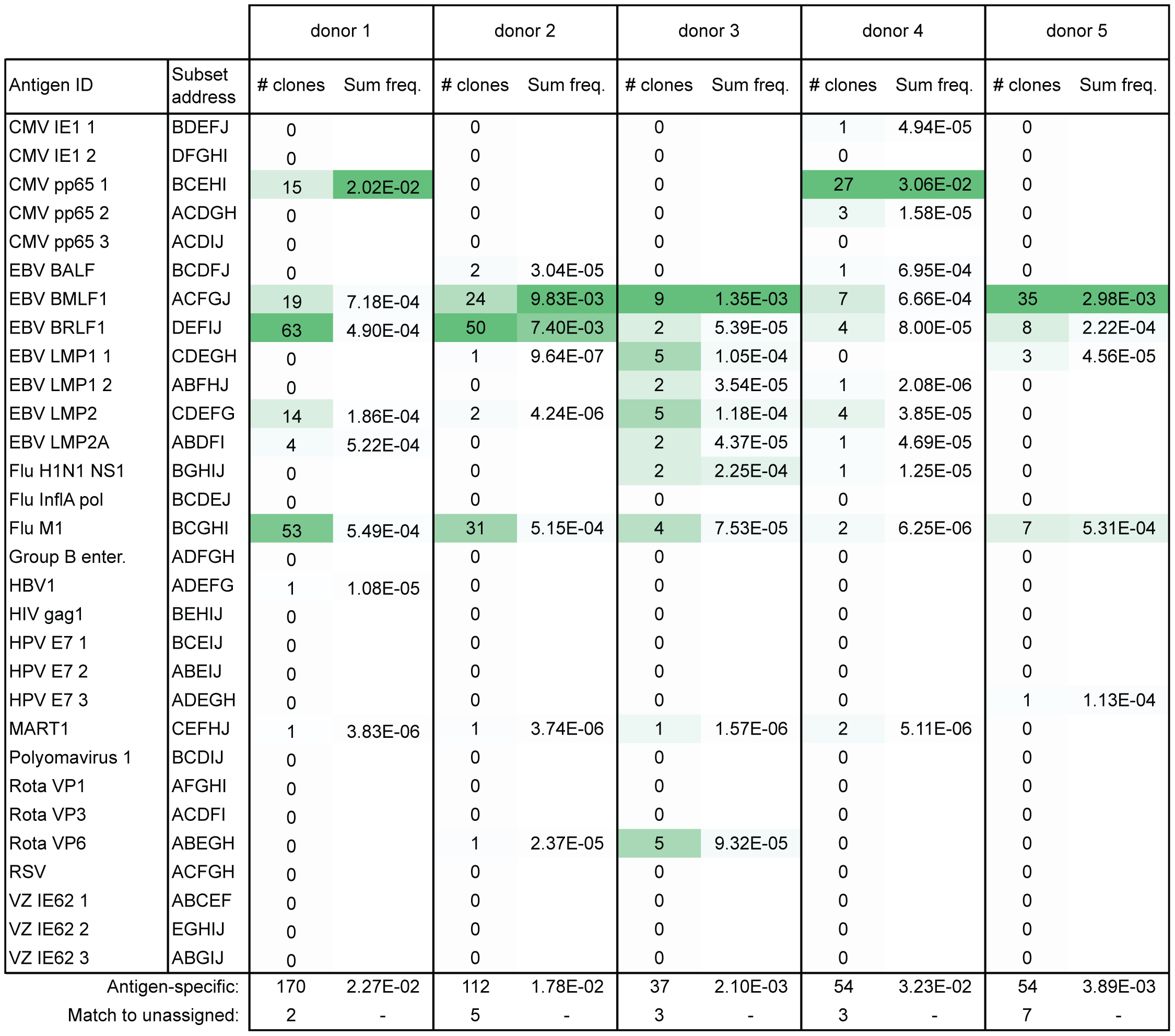

Supplement: S6 Table — (TIF) [file pone.0141561.s013.tif]

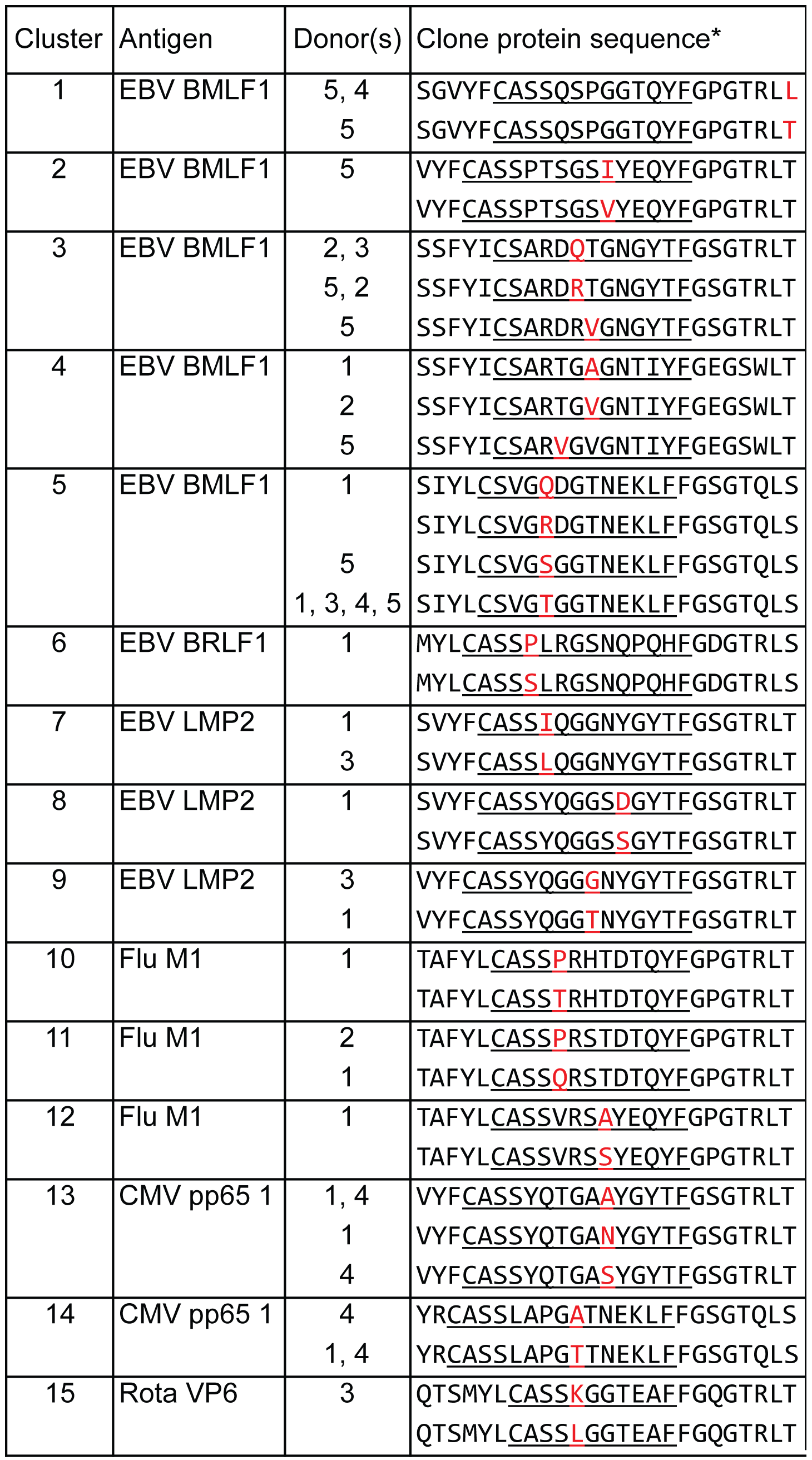

Supplement: S7 Table — 15 clusters of antigen-specific clonotype protein sequences are listed with antigen specificity determination indicated. CDR3 sequences are underlined. (TIF) [file pone.0141561.s014.tif]

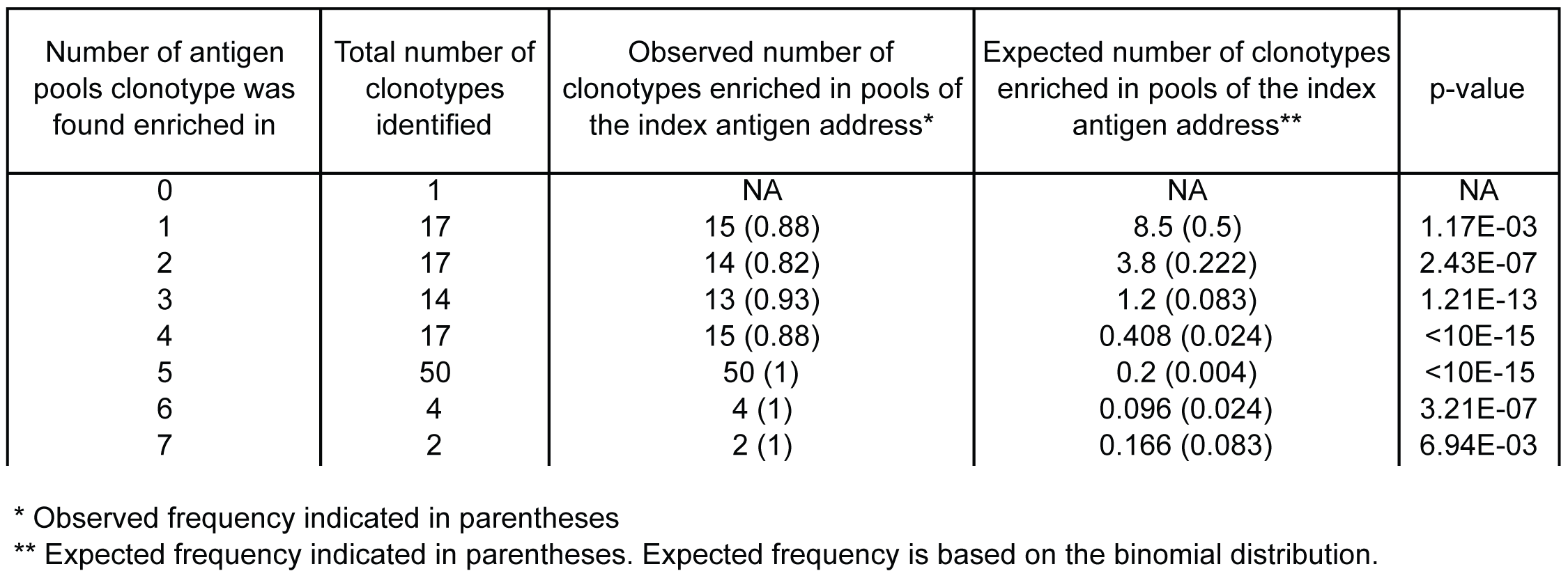

Supplement: S8 Table — The number of clonotypes from an individual matching an antigen-specific query clonotype protein sequence identified in an index individual are indicated in the second column. The observed number of matching clonotypes that were enriched in the positive fraction of at least one of the pools of the expected antigen address of the query sequence are indicated in the third column. The number of matching clonotypes expected to be enriched in the antigen address pools if clonotype enrichment occured randomly are indicated in the fourth column. The computed p-values of observed versus expected events are shown in the last column. (TIF) [file pone.0141561.s015.tif]
